# Cyclists’ exposure to traffic-generated air pollution in multi-modal transportation network design problem

**DOI:** 10.1371/journal.pone.0286153

**Published:** 2023-06-02

**Authors:** Elham Mortazavi Moghaddam, Gholamreza Shiran, Ahmad Reza Jafarian-Moghaddam, Ali Naaman

**Affiliations:** Department of Railway Engineering and Transportation Planning, University of Isfahan, Isfahan, Iran; Jeonbuk National University, REPUBLIC OF KOREA

## Abstract

Moving toward sustainable transportation is one of the essential issues in cities. Bicycles, as active transportation, are considered an important part of sustainable transportation. However, cyclists engage in more physical activity and air intake, making the quality of air that they inhale important in the programs that aim to improve the share of this mode. This paper develops a multi-modal transportation network design problem (MMNDP) to select links and routes for cycling, cars, and buses to decrease the exposure of cyclists to traffic-generated air pollution. The objective functions of the model include demand coverage, travel time, and exposure. The study also examined the effect of having exclusive lanes for bicycles and buses on the network. In the present study, the non-dominated storing genetic algorithm (NSGA-II) solves the upper-level and a method of successive average (MSA) unravels the lower level of the model. A numerical example and four scenarios evaluate the trade-off between different objective functions of the proposed model. The results reveal that considering exposure to air pollution in our model results in a slight increase in travel time (4%) while the exposure to traffic-generated air pollution for cyclists was reduced significantly (47%). Exclusive lanes also result in exposure reduction in the network (60%). In addition, the demand coverage objective function performs well in increasing the total demand in the network by 47%. However, more demand coverage leads to a rise in travel time by 28% and exposure by 58%. The model also showed an acceptable result in terms of exposure to traffic-generated air pollution compared to the model in the literature.

## Introduction

The expansion of cities is a phenomenon that has affected many aspects of urbanization, including transportation. The growth of cities brings many emerging issues in the matter of transportation, namely, traffic congestion, accidents, and environmental issues. To deal with these problems urban mobility is moving toward sustainable transportation. Walking and cycling, known as active transportation, provide safety and environmental benefits in comparison to motorized personal vehicles. However, direct exposure to the environment makes cyclists and pedestrians vulnerable road users. One of the aspects of their vulnerability is their exposure to traffic-generated air pollution. While a person is physically active, breathing and ventilation rates increase, and thus the amount of air pollution inhaled is likely to increase. This can be more problematic for cyclists. Cycling alongside busy roads raises the level of exposure to air pollution. Levels of vulnerability to air pollution can vary depending on which routes are taken; therefore, providing an eligible built environment for active transportation users is one of the options to move toward sustainability [1].

Creating the necessary substructure for urban transportation is known as the urban transportation network design problem [2]. A key hypothesis in these types of problems is the consideration of different transportation modes, which leads to more functional results, especially in real-world problems [3]. These groups of urban transportation network design problems are known as Multi-Modal Network Design Problems (MMNDPs). MMNDPs are usually modelled in two levels. The first level is defined as the decision-maker’s problem. At this level, the choice of the appropriate routes for transportation modes is proposed. While the second level refers to the users’ decisions in which, traffic assignment and model split are customized. Generally at this level, the number of passengers of all modes on routes is calculated [3]. The present study aims to design a multi-modal network with private cars, buses and bicycles by prioritizing decreasing cyclists’ exposure to traffic-generated air pollution.

Most of the MMNPs are in fact transit network design problems that consider transit and private cars interaction at the network level. Meanwhile, there are studies associated with the design of bicycle networks. Mesbah et al. [4] proposed an optimized structure for assigning bicycle lanes to the existing network. Their model’s objective function was to minimize the cyclists’ travel distance and cars’ travel time. They considered the interaction of cars and bicycle transportation modes toward route choice (traffic assignment) at the second level of the model. Doorley et al. [5] proposed a mathematical model to identify the most suitable design for a cycling network to optimize public health (physical activity), environmental impacts (motor vehicles emission), and travel benefits and costs. They presented a multi-modal network with buses, cars, and cyclists. Bagloee et al. [6] MMNDP presented to dedicate the capacity of links to exclusive bicycle lanes. The objective function of the upper-level was minimizing the travel time of cars and cyclists. Lui et al. [7] formalized an optimization model to obtain the best bike route designing plan with the available budget. In this model, only cycling was considered. They used a logit model to calculate the cycling utility of choosing a route, the objective of the model was to maximize this utility. Ospina et al. [8] presented a model that maximizes the coverage of cyclists while maintaining total network cost at its minimum. Liaw et al. [9] proposed a bikeway network design problem in a multi-objective model. The model aimed to minimize risk and impact, and to maximize accessibility and comfort. The interaction of cyclist and other road users were not considered in their model.

In terms of the objective function in bicycle network design, other than the mentioned studies, various studies can be reviewed. Smith et al. [10] proposed the bike network design problem aimed to reduce travel distance and level of service. Zhu et al. [11] presented a bike network design model in that the objective function of the model was accessibility, bicycle level of service, number of intersections, and construction costs. Duthie et al. [12], introduced a network design problem that retrofits existing routes for bicycle infrastructures by minimizing the cost. Luo et al. [13] presented a study on exposure to air pollution for cyclists. The authors estimated the exposure to traffic-generated air pollution for cyclists in the existing routes, and then they presented a routing method to find routes with less exposure for cyclists. The presented model in their study was a routing problem and not a network design problem. Tan et al. [14] also proposed a dynamic system optimal problem that considers the impacts of vehicular emission on human health in the transportation network’s zone. Sun et al. [15] studied the impact of exposure of humans to traffic-generated air pollution on route choice models. According to this background, the factor of exposure to air pollution for cyclists was not studied at the network design level.

The present study is one of the few studies that evaluate exposure to traffic-generated air pollution in the MMNDP for cyclists. Consideration of these factors at the network design level is beneficial in terms of placing bicycle paths that are less exposed to traffic-generated air pollution. This can be particularly valuable in the early stage of using the bicycle network when the shift from motorized vehicles to cycling happens slowly. This study aims to investigate the impact of the objective functions of travel time, exposure for cyclists, and demand coverage in the network design problem’s results. To expand the study, we also evaluate the effect of considering exclusive lanes on the amount of exposure objective function.

MMNDPs are categorized as NP-hard problems, and it is complicated to solve such problems using exact techniques [16]. Most papers regarding MMNDPs have used meta-heuristic methods as a solution algorithm. The genetic algorithm is the most frequently applied algorithm among these methods. For instance, [5, 17, 18] Genetic algorithm was used to solve the bike network design problem. The present paper uses a multi-objective genetic algorithm with a proper mutation and cross-over operation. We used this method since solving the problem as a multi-objective offers a better knowledge of the differences between objectives. Moreover, the popularity of GA makes it more interpretable for planners.

The rest of the paper is structured as follows: The Methodology section defines the bi-level model, the Solution algorithm section presents the applied algorithm for the upper-level and the lower-level, the Results part provides the numerical examples, scenarios definitions and the discussion of the results of the scenarios, and the comparison of the model and a literature model.

## Methodology

### Modelling definition

**Fig 1:**
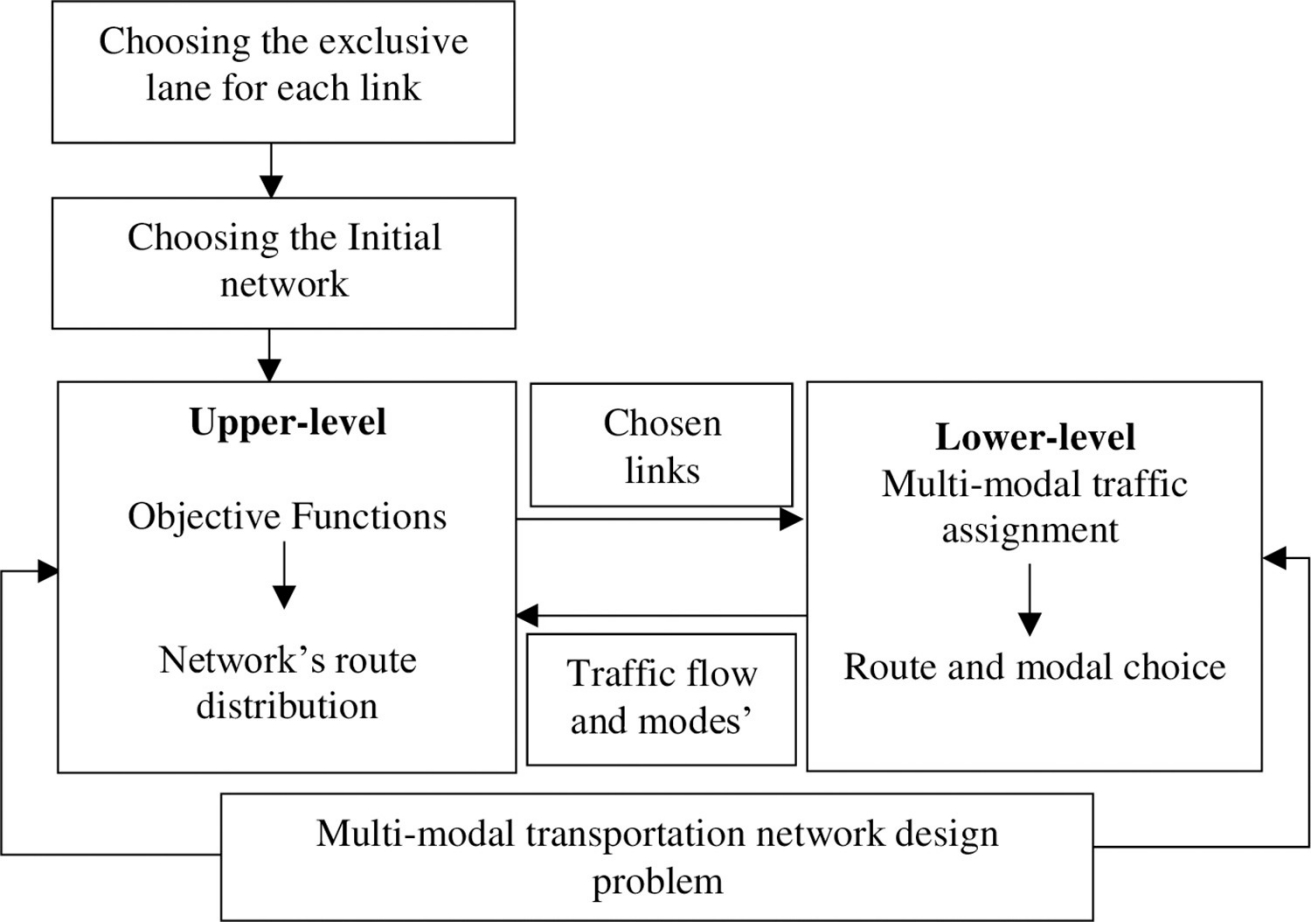
The framework of the bi-level model.

The present study proposes the bicycle network design as a MMNDP, which models the problem in two levels. The first level aims to minimize travel time, reduce exposure to traffic-generated air pollution; and maximize demand coverage. The second level evaluates the interaction between traffic and passenger flow of the different transportation modes [19]. Fig 1 shows the order of levels where the lower-level is solved first and its results are used to solve the upper-level problem.

The combination of bus and bike-exclusive lanes is suggested according to each link’s existing capacity and width. The exclusive lanes are:

1. Shared lanes for all modes
2. On-street lanes for bikes and share lanes for buses and cars
3. On-street lanes for bikes and exclusive lanes for buses
4. Off-street lanes for bikes and shared lanes for buses and cars
5. Off-street lanes for bikes and exclusive lanes for buses

On-street lanes are exclusive lanes that are placed on a street alongside other traffic lanes and are separated by physical barriers. Off-street lanes are exclusive bike lanes separated from the traffic lanes by barriers such as trees.

### Hypotheses

We considered certain assumptions to reduce the complexity and solving time of the model. These assumptions are explained as follows:

1. Travel time consists of in-vehicle or moving time. Stopping time, waiting time and access time are denied.
2. Choosing the proper exclusive lanes for each link is determined before defining the model concerning the width of links.
3. In the presented model capacity of links is not taken into account.

The simplifications both decrease the complexity and, in some cases, help misleading results to be prevented.

### Model development

The mathematical notation for MMNDP is described as follows: Let *G (S*, *A)* be available network’s graph where *S* is sets of Nodes and *A* is sets of links. The summary of the presented model is described as follows:

**Table:** 

**Sets**	
{*i*,*j*}∈*S*	Set of nodes
{*o*,*d*}∈*B*	Set of origins and destinations
*a*∈*A*	Set of links
$r\in R_{od}^{m}$	Set of routes between origin *o* and destination *d*
*m*∈{*c*,*p*,*b*}	Set of transportation modes
*l*∈*L*	Set of public transportation lines
**Indices**	
*P*	Index of public transportation
*B*	Index of bicycle
*C*	Index of car
*K*	Index of exclusive lane type
**Parameters**	
*PC*	Public transportation vehicle’s capacity
*Vc*	Car’s capacity
*F*_max_,*F*_min_	Maximum and minimum frequency of bus
*ϖ*_max_	Load factor for bus
*n*_*a*_	Number of lanes on link *a*
*L*_*a*_	The length of link *a*
$t_{m}^{0}$	Free flow travel time for mode *m*
*q*_*T*_	Total demand in the network
$q_{od}^{m}$	The demand of mode *m* between origin *o* and destination *d*
*θ*	The standard deviation of choosing a vehicle
*ω*_*k*_	The coefficient regarding the impact of exclusive lane type *k* on the exposure function
**Decision variables**	
$f_{r}^{m}$	The number of passengers of mode *m* on route *r*
$y_{a}^{m}$	The number of passengers of mode *m* on link *a*
$x_{a}^{m}$	Traffic flow of mode *m* on link *a*
*Q*_*l*,*max*_	Number of passengers on the busiest link of line *l* for public transport
*F*_*l*_	Bus frequency on line *L*
$int_{a,k}^{m}$	Travel time on link *a* for exclusive lane type *k* for mode *m*
$t_{od}^{m}$	Minimum travel time between origin *o* and destination *d* for mode *m*
*T* ^*m*^	Total travel time for mode m
*CD*	Total covered demand in the network
*EX*	Total exposure to air pollution for the network

#### Multi-modal network introduction

In this subsection, the relationship between traffic flow and passenger flow of each transportation mode is calculated using user equilibrium formulation [20].

The relation between total demand and each transportation mode’s demand between origin “*o*” and destination “*d*” is shown as Eq (1).


$$q_{T}=q_{od}^{c}+q_{od}^{b}+q_{od}^{p}$$ (1)

Eq (2) shows the relation between the number of passengers on the link and the route.


$$y_{a}^{m}=\sum_{\{o,d\}\in B}\sum_{r\in R_{od}^{m}}f_{r}^{m}\times \delta_{r,a}^{od}\;\forall m\in \{c,p,b\}\;\forall a\in A$$ (2)

Also Eq (3) calculates the passengers of each mode and the demand between origin and destination.


$$\sum_{r\in R_{od}^{m}}f_{r}^{m}=q_{od}^{m}\;\forall m\in \{c,p,b\}\;\forall \{o,d\}\in B$$ (3)

Accordingly, the total number of passengers on the present routes between each origin and destination is equal to the demand between them. The evaluation of the traffic flow of each mode on each link depends on the number of passengers of that mode. Eq (4) and Eq (5) indicate the car and bus traffic flow, respectively.


$$x_{a}^{c}=y_{a}^{c}/VC\;\forall a\in A$$ (4)

$$x_{a}^{b}=y_{a}^{b}\;\forall a\in A$$ (5)

Assessing the bus traffic flow is undertaken regarding their frequency. Hence, the assessment of frequency in each line is done by Eq (6) [21].


$$F_{\min}\leq F_{l}=\frac{Q_{l,\max}}{\varpi_{\max}.PC}\leq F_{\max}\;\forall l$$ (6)

The bus traffic flow will be realized according to Eq (7)

$$x_{a}^{p}=\sum_{l}F_{l}\times \sigma_{l,a}\;\forall a\in A$$ (7)

#### Upper-level model

The upper-level problem covers three objectives including travel time, exposure to traffic-generated air pollution, and covered demand. The following subsections explain each objective function.

#### Travel time

Numerous researchers in network design problems have used the Bureau of Public Roads (BPR) model to calculate the flow-dependent travel time [22].


$$t=t_{0}\times [1+\alpha {\left(\frac{Q}{C}\right)}^{\beta }]$$ (8)

In the above equation, *t*_*0*_ is free flow-travel time, *Q* is traffic flow on the path, and *C* is the route’s capacity. α and β are the parameters of the equation.

This equation can determine the travel time concerning capacity and traffic flow on each link. The travel time for each of the exclusive lanes is different (see S1 File). Having the link’s travel time, the total travel time for each mode can be achieved using Eq (9).


$$T^{m}=\sum_{a\in A}\sum_{k}int_{a,k}^{m}\times \eta_{a,k}\times y_{a}^{m}\;\forall m\in \{c,p,b\}$$ (9)

#### Exposure to traffic-generated air pollution

The pollutant factor that is of interest in the present study is Carbon Monoxide (CO). To consider exposure to CO, first, the CO emission should be estimated. Zhang et al. [23]. Proposed a macroscopic model to calculate emission as Eq (10). It shows a liner emission equation (*mg/s*). In Eq (10) the travel time is in terms of hour, and the link’s length is in kilometer.


$$E_{a}=0.2038\times (\frac{x_{a}^{p}+x_{a}^{c}}{3600\times L_{a}})\times ({\mathrm{int}}_{a,k}^{p}+{\mathrm{int}}_{a,k}^{p})\times \eta_{a,k}\times e^{0.7962(L_{a}/({\mathrm{int}}_{a,k}^{p}+{\mathrm{int}}_{a,k}^{p}))}$$ (10)

To evaluate the concentration of emissions from a continuous point source using Eq (11). Which was presented by Turner [24] in the form of a Gaussian equation.


$$C(x,y,z)=\frac{E}{2\pi \sigma_{y}\sigma_{z}u}\exp(-\frac{y^{2}}{2\sigma_{y}^{2}}).[\exp(-\frac{{\left(z-H_{p}\right)}^{2}}{2\sigma_{z}^{2}})+\exp(-\frac{{\left(z+H_{p}\right)}^{2}}{2\sigma_{z}^{2}})]$$ (11)

Where *C(x*,*y*,*z)* represents the mean emission concentration in a point source (*mg/m*^*3*^) with *x*,*y*, and *z* coordinates. *x* and *y* are, respectively, the cross-wind and along-wind coordination. While *z* shows the coordinate of the point source from the ground. *u* is the mean wind speed in meters per second, *E* indicates the strength of the point source in mg per second, and *H*_*p*_ is the elevation of the plume where emissions are released. σ_z_ and σ_y_ are the standard deviation of vertical and horizontal diffusion that depends on the atmosphere respectively.

Since CO emission occurs near the ground surface, *z* and *H*_*p*_ are equal to *0*. Based on this, the Eq (11) is modified as the Eq (12) [23].


$$C(x,y,z=0)=\{\begin{array}{l} 0,x<0\\ \frac{E}{\pi \sigma_{y}\sigma_{z}u}\exp(-\frac{y^{2}}{2\sigma_{y}^{2}}),x\geq 0 \end{array}$$ (12)

The above equation is based on a point emission source; meanwhile, all the factors and objectives in MMNDP are based on link features. Thus, the point emission concentration extends to a line source. To do so, Benson [25], proposed a method that divides each line source into small elements and each element is called the Finite line Source (FLS). Since the points along the equivalent FLS have the same *x* coordinate, the emission concentration that is caused by each element *e* can be assessed through the integral presented in Eq (13).


$$C_{e}(x,y)=\int_{y_{e}^{1}}^{y_{e}^{2}}c(x,y,z=0)dy=\frac{E_{e}}{\pi \sigma_{y}\sigma_{z}u\sin\varphi_{e}}\int_{y_{e}^{1}}^{y_{e}^{2}}\exp(-\frac{y^{2}}{2\sigma_{y}^{2}})dy$$ (13)

Here $y_{e}^{1}$and $y_{e}^{2}$are y coordinate distance between two endpoints of the small element *e*.

Eq (14) can calculate the emission concentration caused by FLS at a location with coordinates (*x*,*y)*. The set of finite lines in the network is presented by F. Therefore, the strength of element *e*, donated as *E*_*e*_, represents the amount of emission in element *e*. If *e* is part of the FLS of link *aϵA* (*e*∈*F*_*a*_), then *E*_*e*_ *= E*_*a*_.


$$C_{e}(x,y)=\frac{\sqrt{2}E_{e}}{\pi \sigma_{y}\sigma_{z}u\sin\varphi_{e}}[\phi (\frac{y_{e}^{2}}{\sigma_{y}})-\phi (\frac{y_{e}^{1}}{\sigma_{y}})]$$ (14)

In Eq (14), *ϕ*(.) is the cumulative probability density function of a standard normal distribution. Having the emission concentration of CO for each link, the exposure to traffic-generated air pollution for cyclists can be calculated according to travel time and type of exclusive lanes as in Eq (15).


$$EX_{a}=\frac{int_{a,k}^{c}\times \eta_{a,k}\times \omega_{k}}{\left|F_{a}\right|}.\sum_{s\in F_{a}}C_{s}(x_{s},y_{s})$$ (15)

Eq (15) shows the exposure to air pollution on link *aϵA* in milligrams, passenger, minute per m^3^. *C*_*s*_ is the emission concentration in element *s* with coordinates (*x*_*s*_, *y*_*s*_). *F*_*a*_ is the set of finite lines of link *aϵA*. The travel time is in minutes.

#### Demand coverage

The demand coverage objective maximizes the network’s accessibility for total demand by maximizing the number of covered demands.


$$CD=\sum_{a\in A}\sum_{k}y_{a}^{m}\times \eta_{a,k}$$ (16)

The upper-level problem through three objectives is as follows:

$$\mathrm{Min}(T^{p}+T^{b}+T^{c}+T^{w})/\sum_{\{o,d\}\in B}q_{od}$$ (17)

$$\mathrm{Min}\;EX=\sum_{a\in A}EX_{a}$$ (18)

MaxCD (19)

Eq (17) minimizes the average travel time per passenger. Eq (18) minimizes total exposure to air pollution. Eq (19) refers to maximizing the covered demand in the network.

#### Lower-level

The lower-level is the multi-modal User Equilibrium (UE). The model assesses traffic interaction of various modes in terms of traffic assignment and mode choice. The results of this level are the equilibrium of MMNDP including traffic flow and passenger flow on each route and demand for each transportation mode.

#### Lower-level’s objective function

Traffic assignment and modal split are measured based on travel time in the UE model. Thus, the travel time in each route is as Eq (20).


$$t_{r}^{m}=\sum_{a\in A}\sum_{k}int_{a,k}^{m}\times (\eta_{a,k}\times \delta_{r,a}^{od})\;\forall r\in R_{od}^{b}\;\forall m\in \{c,p,b\}$$ (20)

For the mentioned travel modes, passengers will choose a route that minimizes their travel time. Eqs (21) to (24) demonstrate the equilibrium of the network [20].


$$(t_{r}^{m}-t_{od}^{m})\times f_{r}^{m}=0\;\forall m\in \{c,p,b\},\forall r\in R_{od}^{m},\forall \{o,d\}\in B$$ (21)

$$f_{r}^{m}\geq 0\;\forall m\in \{c,p,b\},\forall r\in R_{od}^{m}$$ (22)

$$t_{r}^{m}-t_{od}^{m}\geq 0\;\forall m\in \{c,p,b\},\forall r\in R_{od}^{m},\forall \{o,d\}\in B$$ (23)

$$\sum_{r\in R_{od}^{m}}f_{r}^{m}=q_{od}^{m}\;\forall m\in \{c,p,b\},\forall od\in OD$$ (24)

Eq (25) shows the modal split using a logit model [20].


$$q_{od}^{m}=q_{od}\times \frac{\exp(-\theta \times t_{od}^{m})}{\exp(-\theta \times t_{od}^{c})+\exp(-\theta \times t_{od}^{p})+\exp(-\theta \times t_{od}^{b})}\;\forall m\in \{c,p,b\}$$ (25)

#### Traffic assignment model constraints

The UE model of the multi-modal network is formulated by Eqs (1) to (7) and Eqs (20) to (24). Thus, it can be written as a mathematical model shown in Eq (26) [26]:

$$\mathrm{Min}\sum_{r\in R_{od}^{m}}\sum_{od\in OD}\sum_{m}(t_{r}^{m}-t_{\min,od}^{m})\times f_{r}^{m}+{\sum_{od\in OD}\left(q_{od}^{m}-q_{od}/1+\sum_{m\in \{c,p,b\}}\exp(\theta \times t_{od}^{m})\right)}^{2}$$ (26)

S.t.

(1) to (7) and (22) to (25)

## Solution algorithm

The problem in the present paper was modelled through MMNDP which is a bi-level problem. These types of problems with accurate procedures are very difficult to solve. The easiest bi-level problems, even though they are linear at both levels, are taken as NP-hard models [27].

The present study used the most popular reputation methods to solve the bi-level model. Multi-objective Non-dominated Sorting Genetic Algorithm (NSGA-II) is one of the most favourable algorithms for the evaluation of multi-objective models [28]. Therefore, to solve the upper-level problem the NSGA-II is used. The lower-level is solved using the Method of Successive Averages (MSA). This method has been extensively used for solving UE models.

### Upper-level algorithm

The basis of the NSGA-II algorithm generates a solution population through consecutive iterations; modifying the solutions with cross-over and mutation operators in each iteration. These operators use a combination of solution and random changes to generate the offspring populations respectively. The solutions and population of offspring are then evaluated and ranked based on two measures: non-dominance and crowding distance sorting. The non-dominance sorting measure assigns the highest rank to the solution that is not dominated by any other response, while the crowding distance sorting method deals with differences between the response and those neighbouring it [29].

For the problem to be in the right direction, we used an Initial Network Selection algorithm (INS) Jha et al. [30] proposed this algorithm. In this algorithm, the initial network is selected according to the width of links for the bicycle’s exclusive lane and the demand between nodes. The INS algorithm follows these steps:

An initial setting before the algorithm is to assign the exclusive bus and bike lane to links based on the links’ width and space.

1. Count the number of links connected to each node, let *Ns* be the number of links connected to node *s*
2. Sort nodes based on the number of connected links in increasing orders
3. Select a subset of *k* nodes based on the following rule:

$$k=\left\{ \begin{array}{l} 2R\;R<N/2\\ N\;otherwise \end{array}\right.$$

*R* is the number of routes

*N* is the total number of nodes in the main network

4. Calculate the probability of each node in the selected subset as follow:

$$P_{n}^{IS}=\frac{a_{n}}{\sum_{n\in IS}a_{n}}$$

*a*_*n*_ is the number of neighbouring nodes of *s*^*th*^ node

*IS* is the Initial node Set

5. Select the first node of the road randomly based on the $P_{n}^{IS}$ probability

After finding the first node of all routes, to select the other nodes of the route follow these set of steps:

1. Calculate the probability of $P_{n}^{NS}$ for each neighbouring node of the previous node

$$P_{n}^{NS}=\frac{b_{n}}{\sum_{n\in NS}b_{n}}$$

*b*_*n*_ is the demand between the previous node and the *s*^*th*^ node

*NS* is a Neighbouring node set

2. Determine the next node randomly based on the $P_{n}^{NS}$ Probability
3. Continue steps 1 and 2 until the termination criteria are met. The termination criteria for INS are (1) reaching the maximum number of nodes in a route, (2) the cardinality of a neighbouring node set of the previous node falls to zero

**Fig 2:**
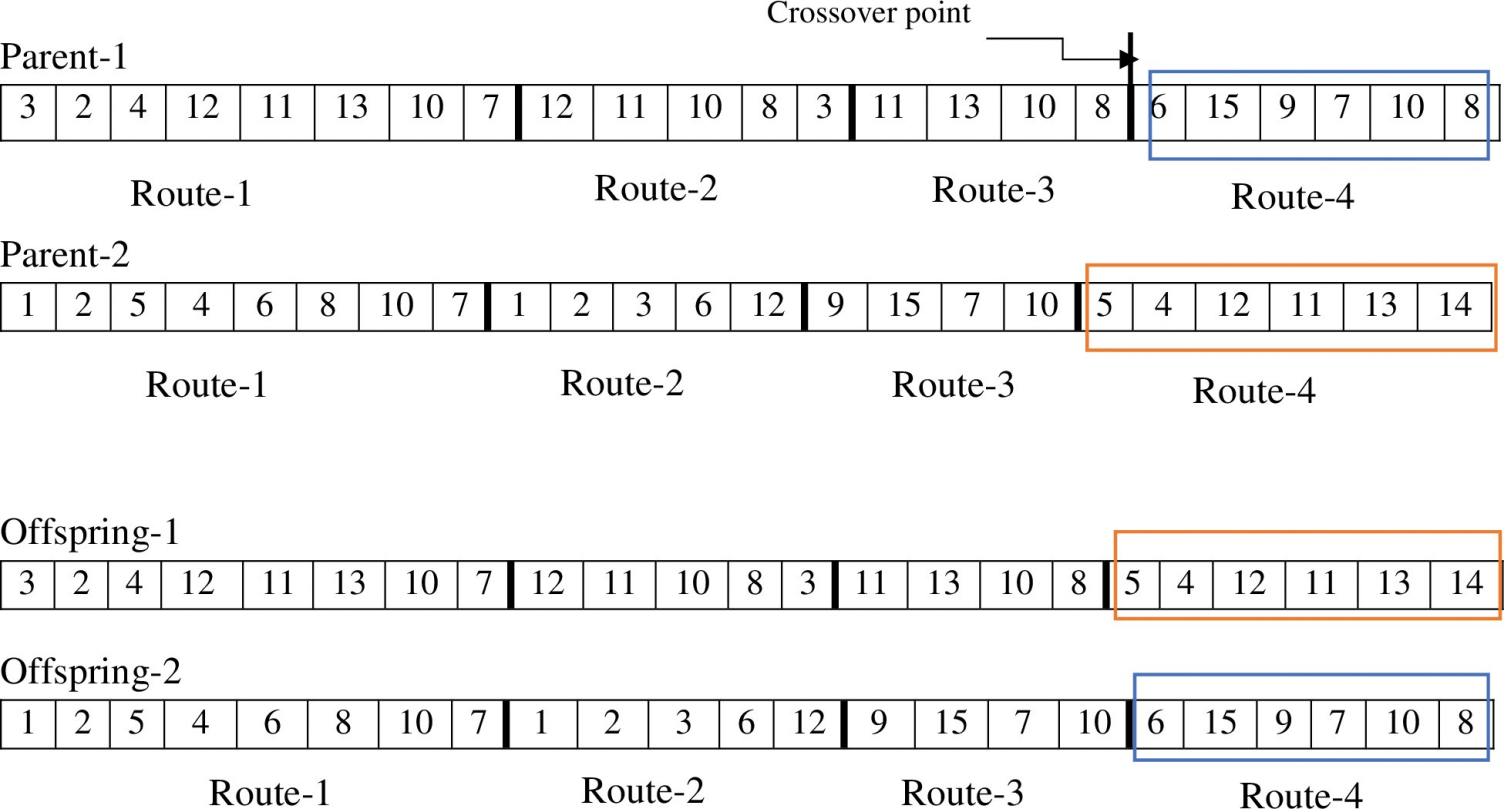
Inter-string cross-over.

**Fig 3:**
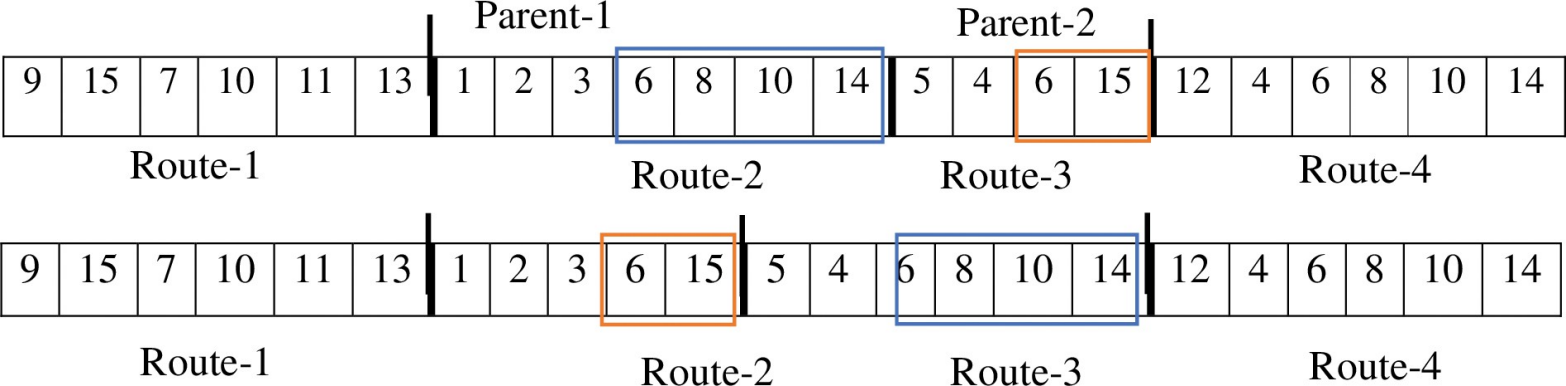
Intra-string cross-over.

To find an optimal solution, the algorithm generates a new population of offspring in each iteration by using appropriate cross-over and mutation operators. The aim of cross-over is an exchange of genes between two chromosomes that lead to creating offspring with better characteristics. Depending on the nature of the problem and the chromosomes, there are two types of cross-over: inter-string cross-over and intra-string cross-over. Inter-string cross-over is used at the route level by exchanging the existing route in parents’ chromosomes, and in intra-string cross-over the solutions are yielded at the level of the nodes [30]. Therefore, two routes from each parent are randomly chosen and then transferred from a common node of their genes. Figs 2 and 3 illustrate the stages of inter-string and intra-string cross-over.

**Fig 4:**
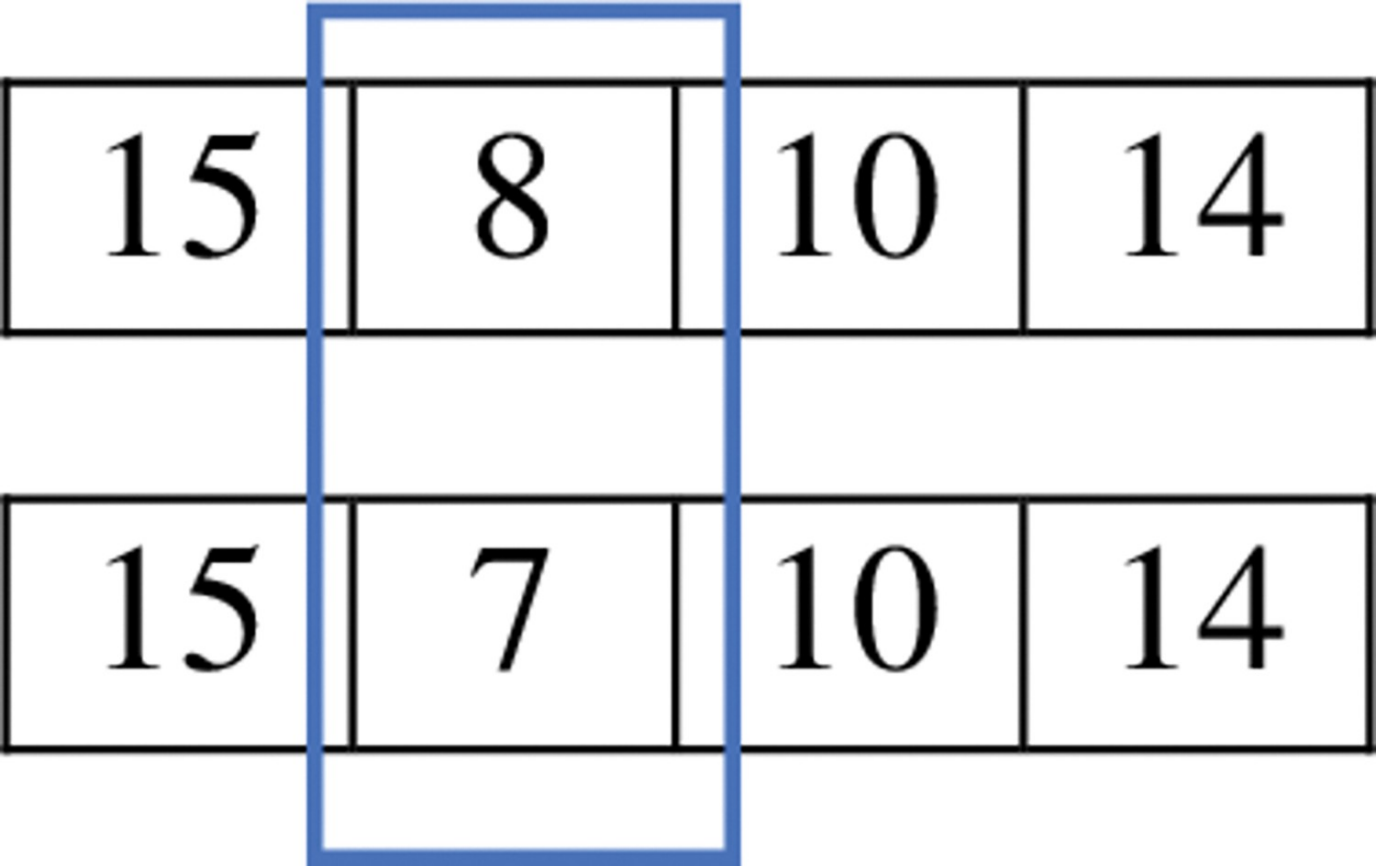
Mutation operator.

The mutation operator aims to agitate the chromosomes by randomly choosing a node from the routes. The probability of selecting a node for mutation is known as the mutation probability. Fig 4 provides an example of a mutation. In this stage, a random node is selected (node 8). Next, a node is chosen (node 7) from the adjacent nodes (nodes 9, 6, 7, and 8) of the previous one (node 15), and it is replaced with the selected one (node 8). It is then verified that the new node would have contact with the next node; otherwise, an additional node can be inserted between the two nodes to keep the route connected.

### Lower-level algorithm

MSA efficiently solves the traffic assignment and modal split [19]. Each iteration in this method, assesses the share of each travel mode, accounting for the least trip costs, using the logit model. Then, the traffic demand follows the shortest route of each mode. Finally, the routes’ flow is updated using successive averaging [20]. The algorithm ceases when the presented solution and equilibrium state solution converge or the algorithm reaches the maximum number of iterations [Ibid]. The steps of the algorithm performed in the current paper are similar to those introduced by [19]:

*Inputs*: the transportation network includes links, nodes, origin-destination matrix, and other parameters. The number of iterations is shown by (*n*), and the convergence coefficient is (*ε*).

*Outputs*: the origin and destination demands of each mode and the number of passengers on each route.

1. Initialization:

The total demand for each mode of transportation ($q_{od}^{m}$) is assumed 0. Besides, for each route between an origin and a destination, the total number of passengers of each mode ($f_{r}^{m}$) will be calculated using Eqs (4), (5), and (7). (*n = 1*)

2. All-or-nothing assignment:

First, the algorithm calculates the travel time for each mode using Eq (9). Then the algorithm selects the minimum travel time between each origin and each destination. Following this, for each origin and destination, the algorithm assesses the demand of each travel mode using the logit model shown in Eq (21). The origin-destination demand of other routes rather than the shortest route will be *0*.

3. Update route flow using MSA

In this step, the passenger flow is determined by weight averaging of “$f_{r}^{m}$” of the previous iterations. Then the $q_{od}^{m}$ is updated through Eq (27).


$$r\in R_{od}^{m}:f_{r}^{m}=\frac{n-1}{n}\times f_{r}^{m}+\frac{1}{n}\times temp^{m}(r)\;\forall m\in \{c,p,b\}$$ (27)

$r\in R_{od}^{m}$: Calculate $q_{od}^{c},q_{od}^{p},q_{od}^{b}$ using Eq (25)

4. Convergence assessment:

$$\sum_{m\in \{c,p,b\}}\frac{\sqrt{\sum_{\{o,d\}\in B}{\left(q_{od}^{m}-{\tilde{q}}_{od}^{m}\right)}^{2}}}{\sum_{\{o,d\}\in B}Q_{od}}+\sum_{m\in \{c,p,b\}}\frac{\sum_{\{o,d\}\in B}\sum_{r\in R_{od}^{m}}(t_{r}^{m}-t_{od}^{m})\times f_{r}^{m}}{\sum_{\{o,d\}\in B}t_{od}^{m}\times q_{od}^{m}}\leq \varepsilon$$ (28)

**Fig 5:**
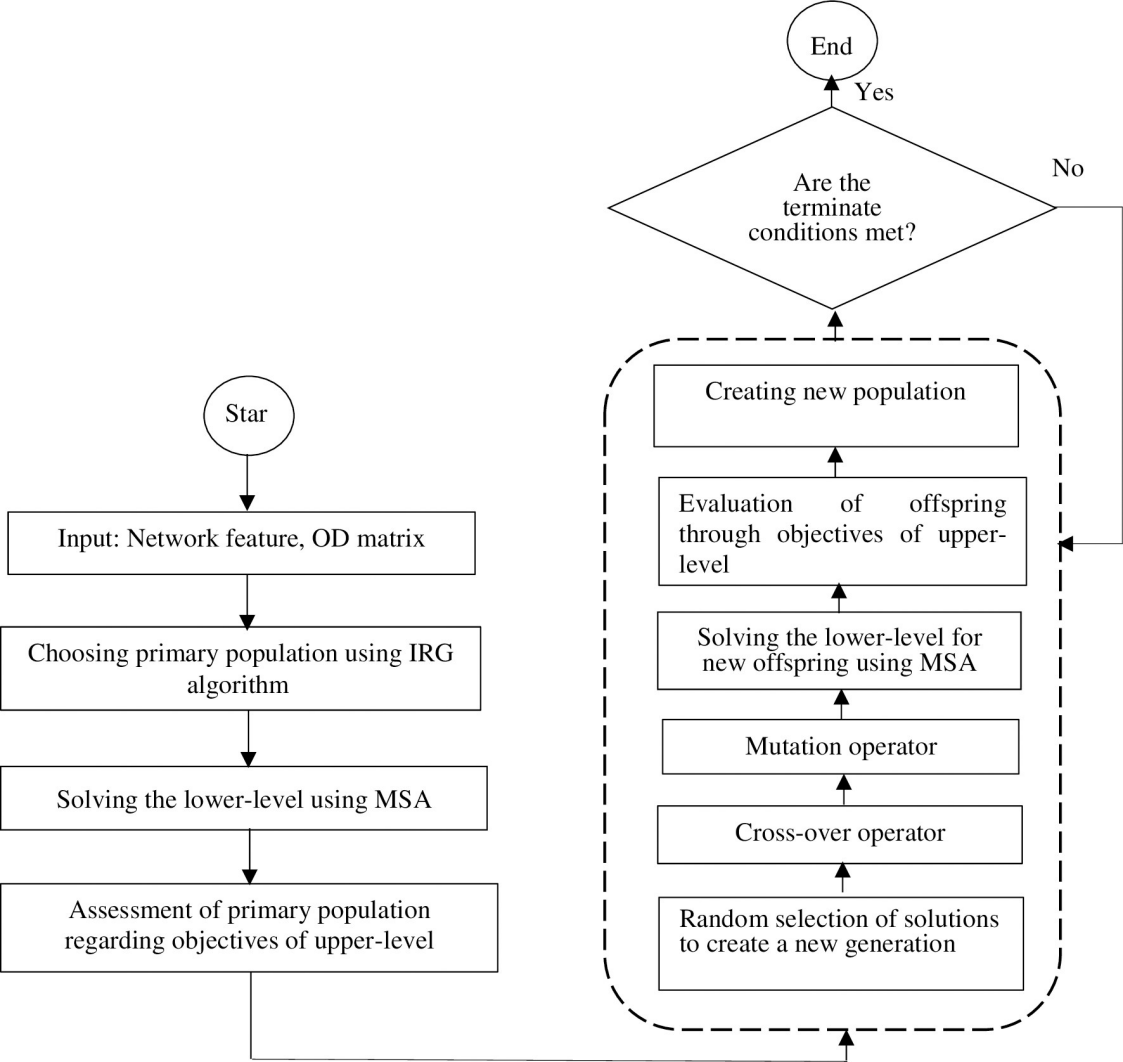
MMNDP flowchart.

If Eq (28) which shows the condition of the convergence is realized, or the number of iterations reached the permissible limit the cycle will cease; otherwise, *n = n+1* and returns to step 2. Fig 5 demonstrates the general structure of the phases of solving the bi-level model.

## Results

### Numerical examples

Numerical experiences were conducted to analyze the features of the developed MMNDP. Two networks were adapted in this section: 1. Mandle’s network; 2. A small network consists of nine nodes and twelve links.

**Fig 6:**
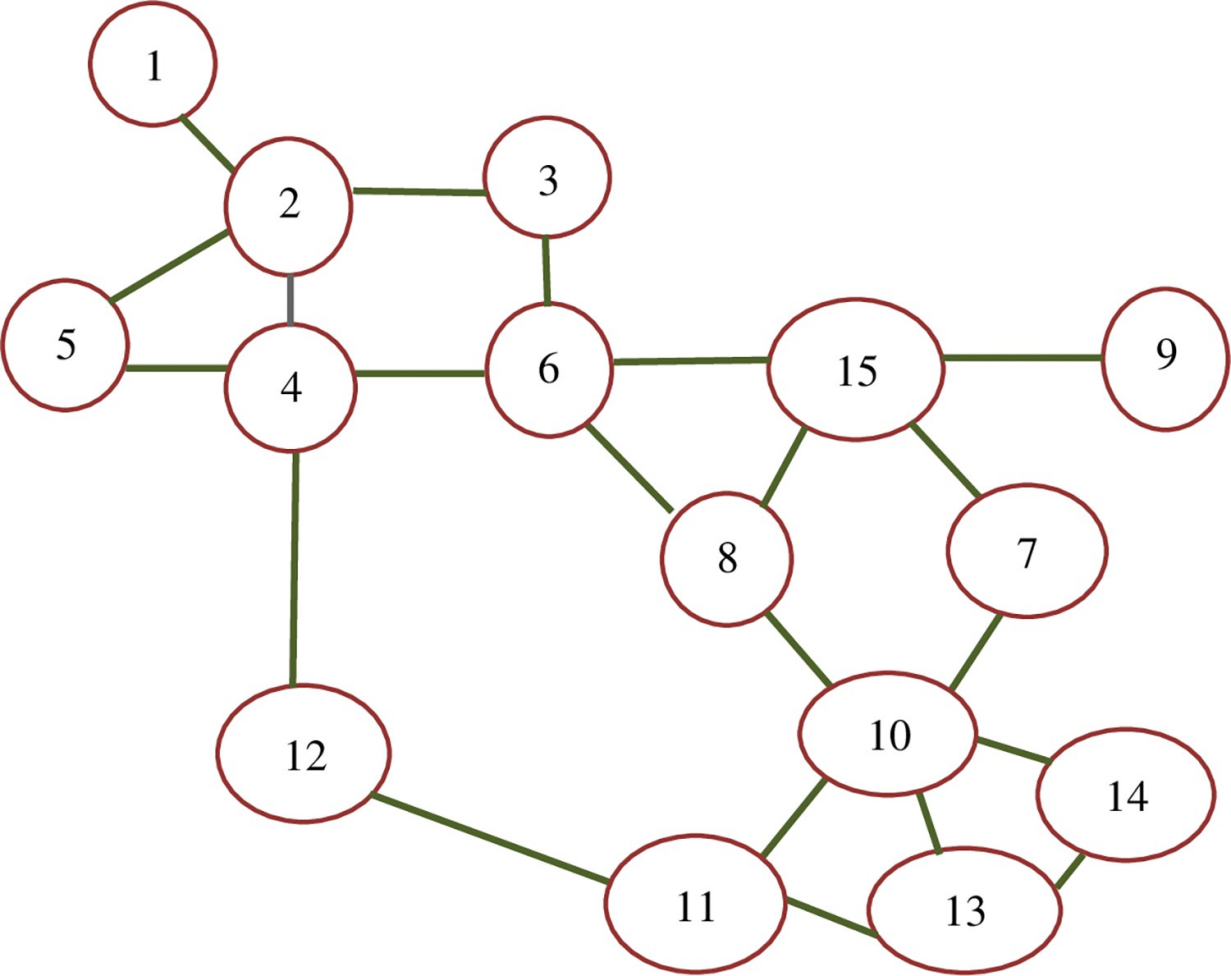
Mandle’s network.

The first network is known as “Mandle’s Swiss road network” which is demonstrated in Fig 6. The network has 15 nodes, 21 links, and a total demand of 15600, and it was first used by Mandl [31] It is a widely adopted test network in the transportation network design field, to show the performance of the models and algorithm. In the presented study, Mandle’s network was used to evaluate the proposed model and test different scenarios related to the objective function of the model.

**Fig 7:**
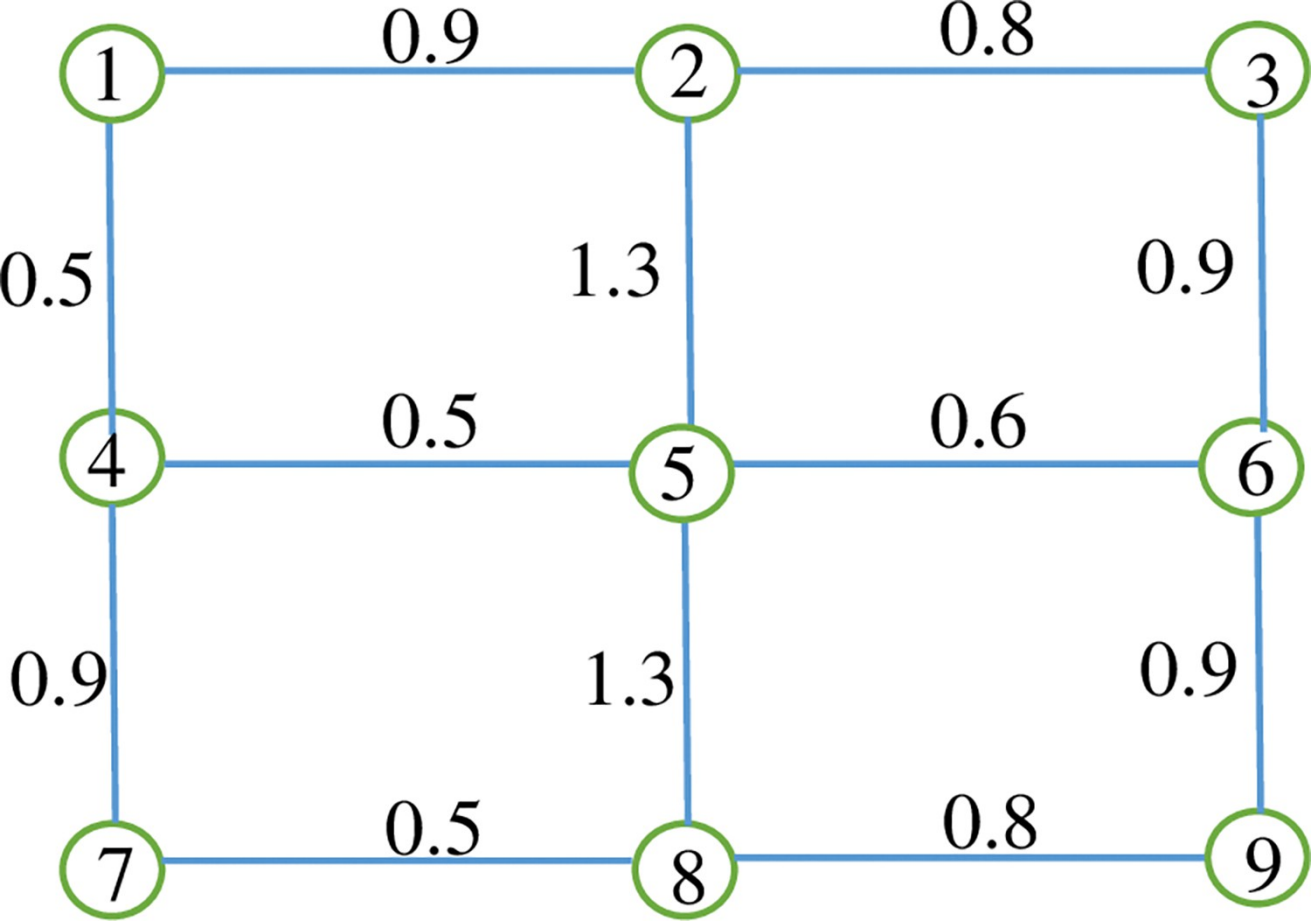
The nine-node network.

The second network is a small network that is shown on Fig 7. The length of the links is presented on Fig 7. The lengths are per *Km*. This network is used to show the differences in the results between our model and that of a bicycle network design model from the literature.

### The Mandle’s network

We presented a MMNDP to design a cycling network that reduces cyclists’ exposure to traffic-generated air pollution. The scenarios examined in this section are as follows:

1. Three-objective model including travel time, exposure, and covered demand, with exclusive bike lanes and bus lanes
2. Three-objective model without exclusive lanes
3. Two-objective model including travel time and exposure with exclusive lanes
4. Two-objective model including travel time and covered demand with exclusive lanes

We presented these scenarios in accordance with the main research aims that have been mentioned in previous sections. To evaluate each scenario, we ran the model 5 times. Three solutions from all the runs were chosen including Minimum Travel Time (Min-TT), Minimum Exposure (Min-Ex), and Maximum Covered Demand (Max-CD). The results of the three most acceptable answers from 5 runs in each scenario are presented in Table 1. According to this table, adding the exposure objective function and considering exclusive lanes in the network, will result in a lower amount of exposure for cyclists. Details of these changes were studied in the following subsections.

**Table 1:** Best answers for each objective function in each scenario.

Scenario	Min -TT			Min -Ex			Max- CD		
	ATT (min/pas)	EX (pas.min.mg/m^3^)	CD (pas)	ATT (min/pas)	EX (pas.min.mg/m^3^)	CD (pas)	ATT (min/pas)	EX (pas.min.mg/m^3^)	CD (pas)
1	10.7	5.7	2250	10.7	5.7	2250	22	19.7	5608
2	16.7	16.1	3732	18.5	13.5	2970	29	20.6	5335
3	9.37	4.97	2438	13.06	3.26	2650	13.06	3.26	2650
4	11.5	23.3	2846	15.5	16.3	4694	20.1	27.2	6878

Note that in the following subsections the x-axis of the graphs represents the number of selected answers for each scenario (15 selected answers).

### Effects of exclusive lanes

**Fig 8:**
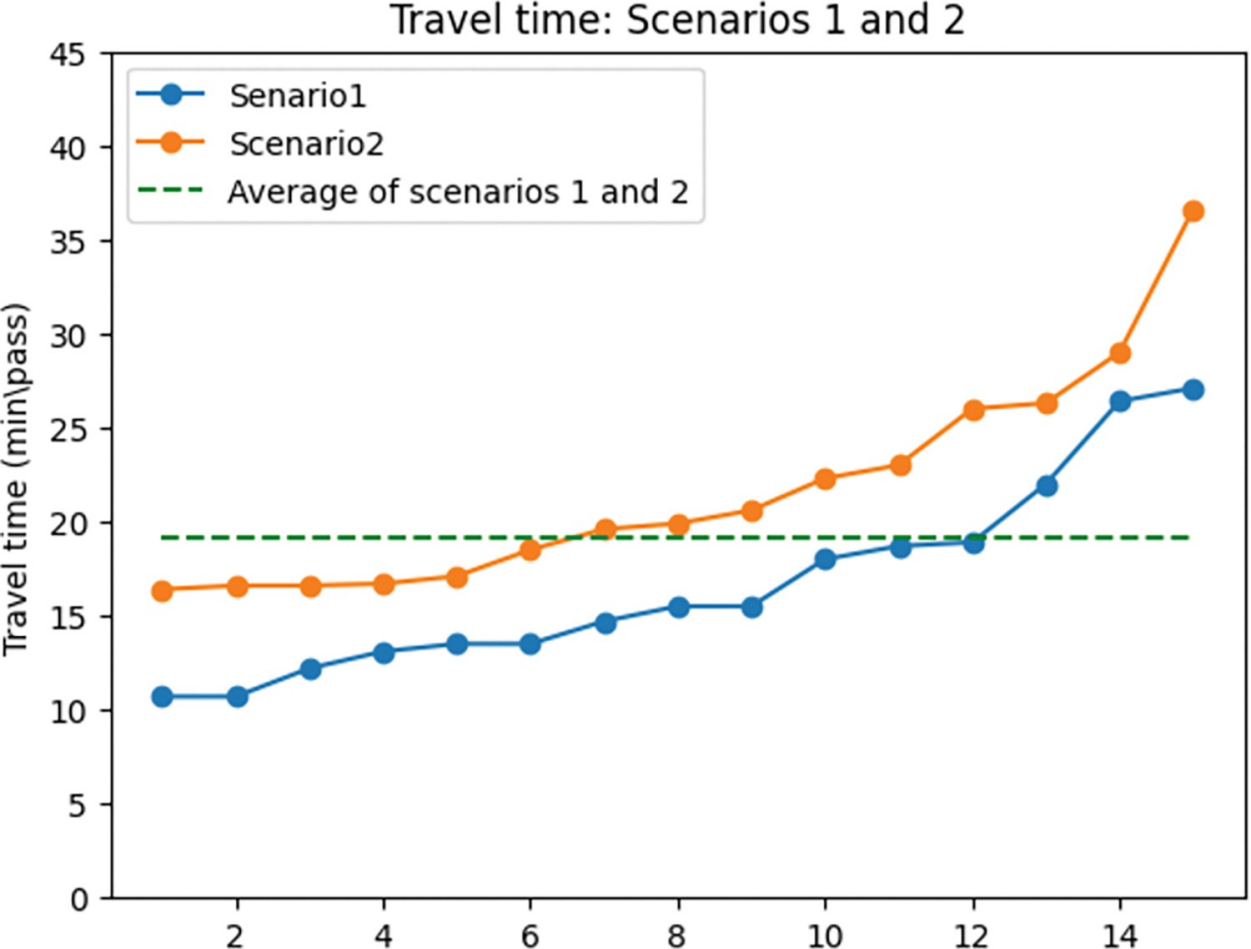
Travel time comparison between scenarios 1 and 2.

To illustrate the effects of exclusive lanes, we compared scenarios 1 and 2. The following observations can be made here:

The first observation is the impact of exclusive lanes on travel time. Fig 8 displays the shifts in travel time in all the 3 selected answers from the 5 runs of the model in scenarios 1 and 2. The green line shows the average of the total travel time in both scenarios. In most cases, the total travel time in the second scenario is more than the average in the graph. To assess the general impact of travel time the average of the 15 selected answers was compared and as expected, the travel time increased by 25.9% in the network with exclusive lanes.

**Fig 9:**
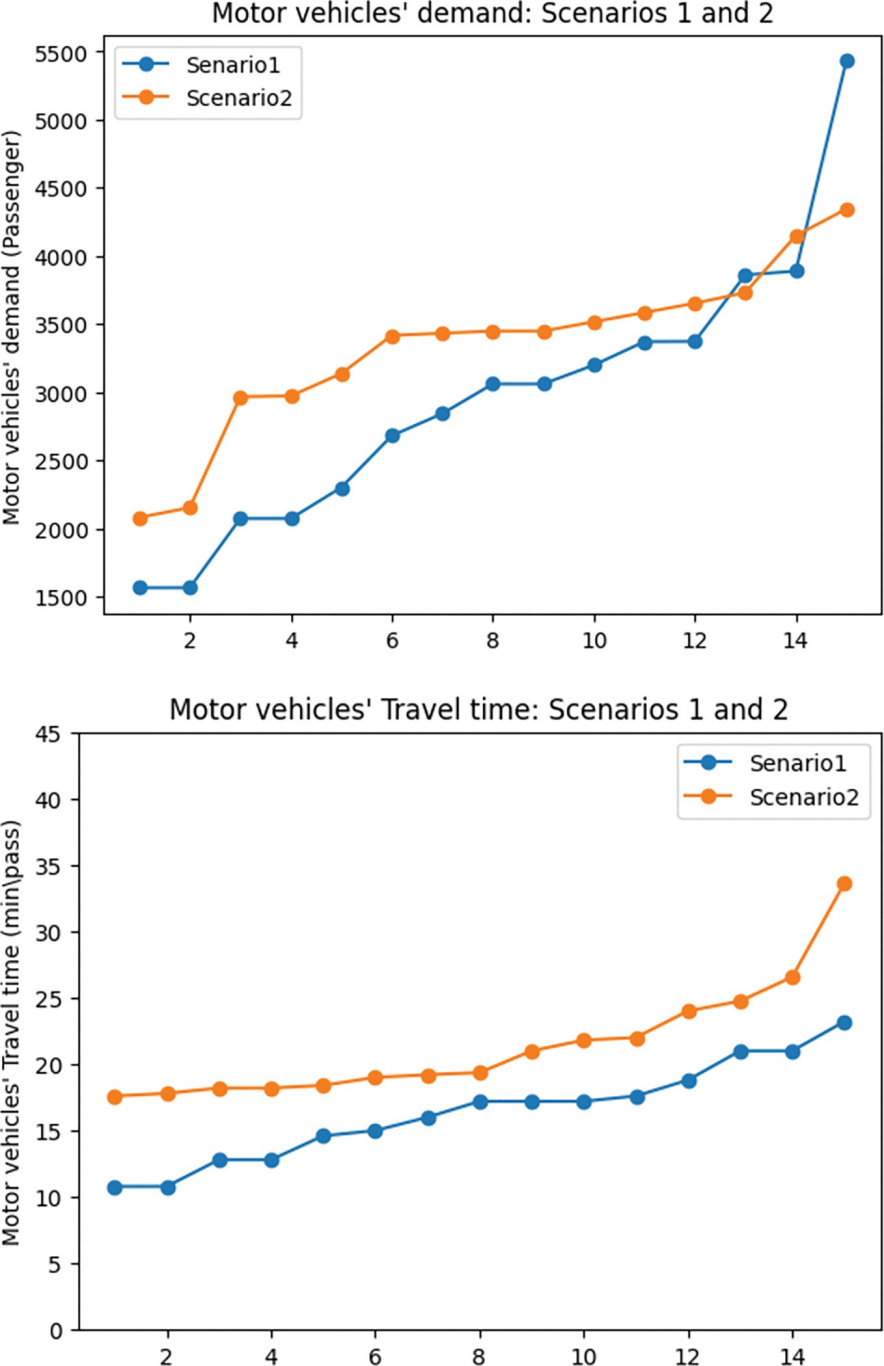
Motor vehicles’ demand and travel time in the scenarios 1 and 2. a) Motor vehicles’ covered demand: scenarios 1 and 2. B) Motor vehicles’ travel time: scenarios 1 and 2.

**Fig 10:**
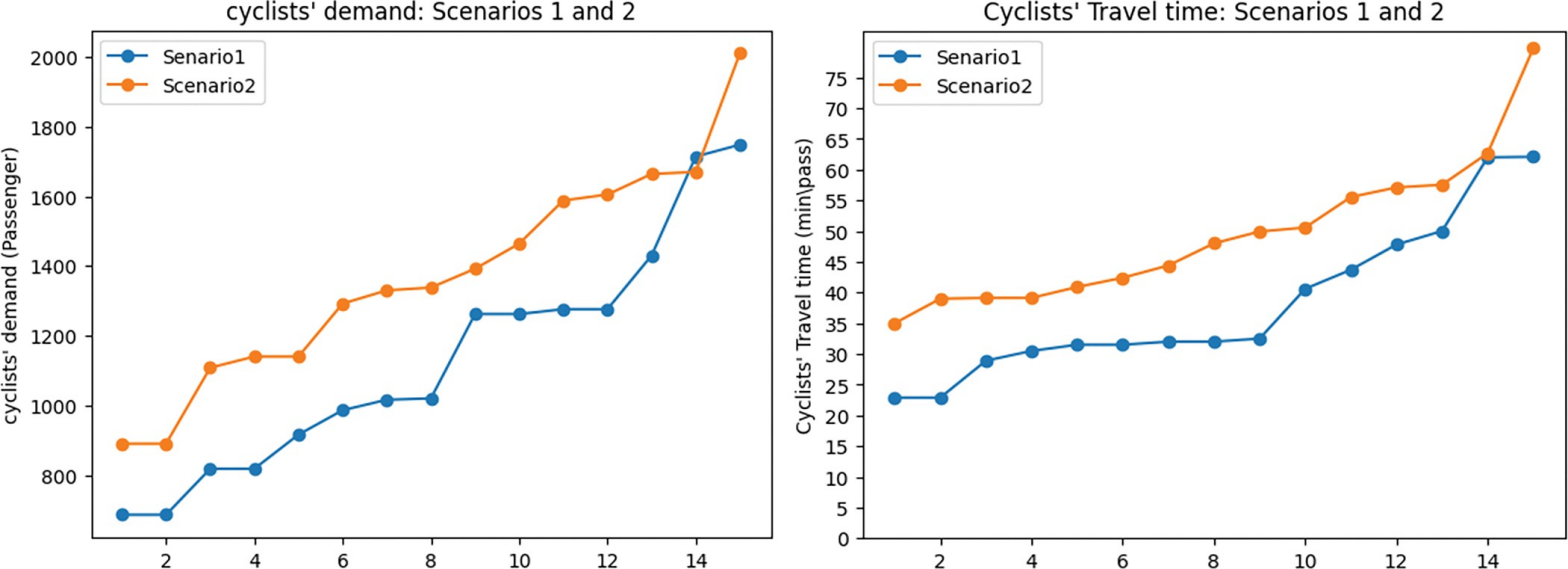
Cyclists’ demand and travel time in the scenarios 1 and 2. a) Cyclists’ covered demand: scenarios 1 and 2. b) Cyclists’ travel time: scenarios 1 and 2.

The reason for this contrast is twofold: First, assigning exclusive lanes to the networks’ links will result in the reduction of road capacity for cars. As in Fig 9A, the average motor vehicle demand in the first scenario is 12% lower than in the second scenario. The reason for this is that the capacity of the road for motor vehicles in the first scenario was assigned to the exclusive lanes. The rise in motor vehicle demand results in a rise in travel time by 26.6%. This trend can be observed in Fig 9B. As Fig 10A shows, despite what is expected, the cyclists’ demand also increased in the second scenario. This happened as sharing the road means that cyclists are able to use all the lanes of the road which results in an increase in the capacity of the road for this mode of transportation. However, it does not prove that omitting exclusive lanes will lead to a rise in cyclists’ demand. In fact, because of safety and comfort issues, cyclists’ demand decreases on shared roads. To show this in the modelling a penalty can be considered for share roads for cyclists’ demand to model the reality better. As a result of the rise in cyclists’ demand and motor vehicles’ demand on shared roads, it can be observed that cyclists’ travel time also increased by 25.9%. Fig 10B shows the cyclists’ travel time trend in scenarios 1 and 2.

**Fig 11:**
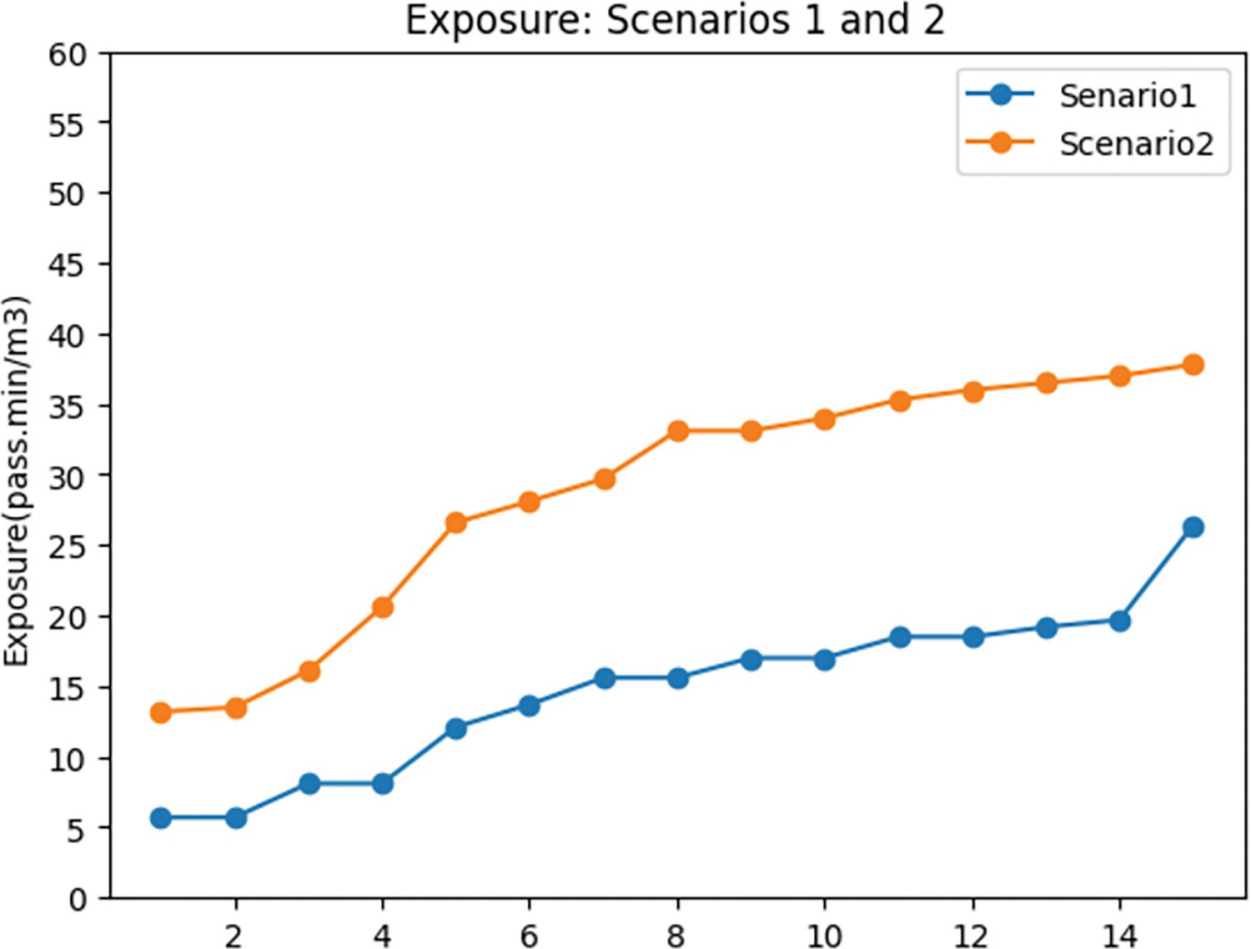
Exposure to traffic-generated air pollution comparison between scenarios 1 and 2.

The second observation regarding the effects of exclusive lanes is on the exposure objective. As Apparicio et al. [32], exposure to traffic-generated air pollurion for cyclists decreases while riding along exclusive lanes. According to this information we considerd a penalty for shared road in terms of exposure to traffic-genenrated air pollution. Fig 11. indicates that the exposure objective increased by 60% in the second scenario, which is consistent with the rise of motor vehicles’ demand in this scenario as in Fig 9.

Please note that the travel time of each mode is calculated by dividing it by the total demand covered by that particular mode. However, to calculate the total travel time of the network, the sum of the travel time of cyclists and motor vehicles is divided by the total demand covered by the network. This approach is used to ensure a fair comparison of the travel time between different modes of transportation.

### Effects of demand coverage

Demand coverage is one of the most frequent objective functions in the network design problems for cyclists. In this subsection, we are planning to evaluate the effects of this objective function on different aspects of the presented network design problem.

To evaluate the impact of demand coverage objective in the network design problem, scenarios 1 and 3 were compared. The results of this contrast are twofold: travel time and exposure to traffic-generated air pollution.

**Fig 12:**
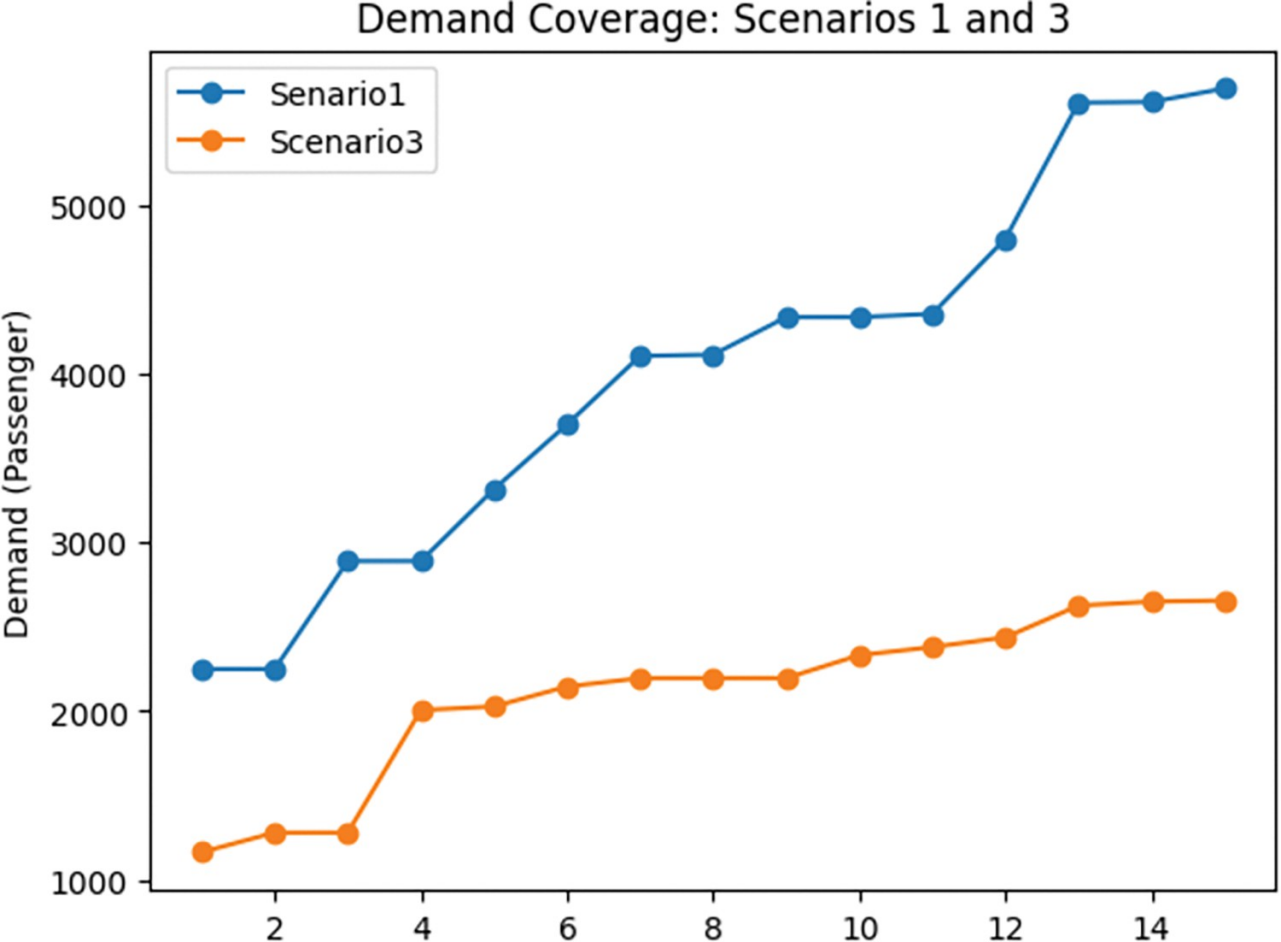
Demand coverage comparison between scenarios 1 and 3.

**Fig 13:**
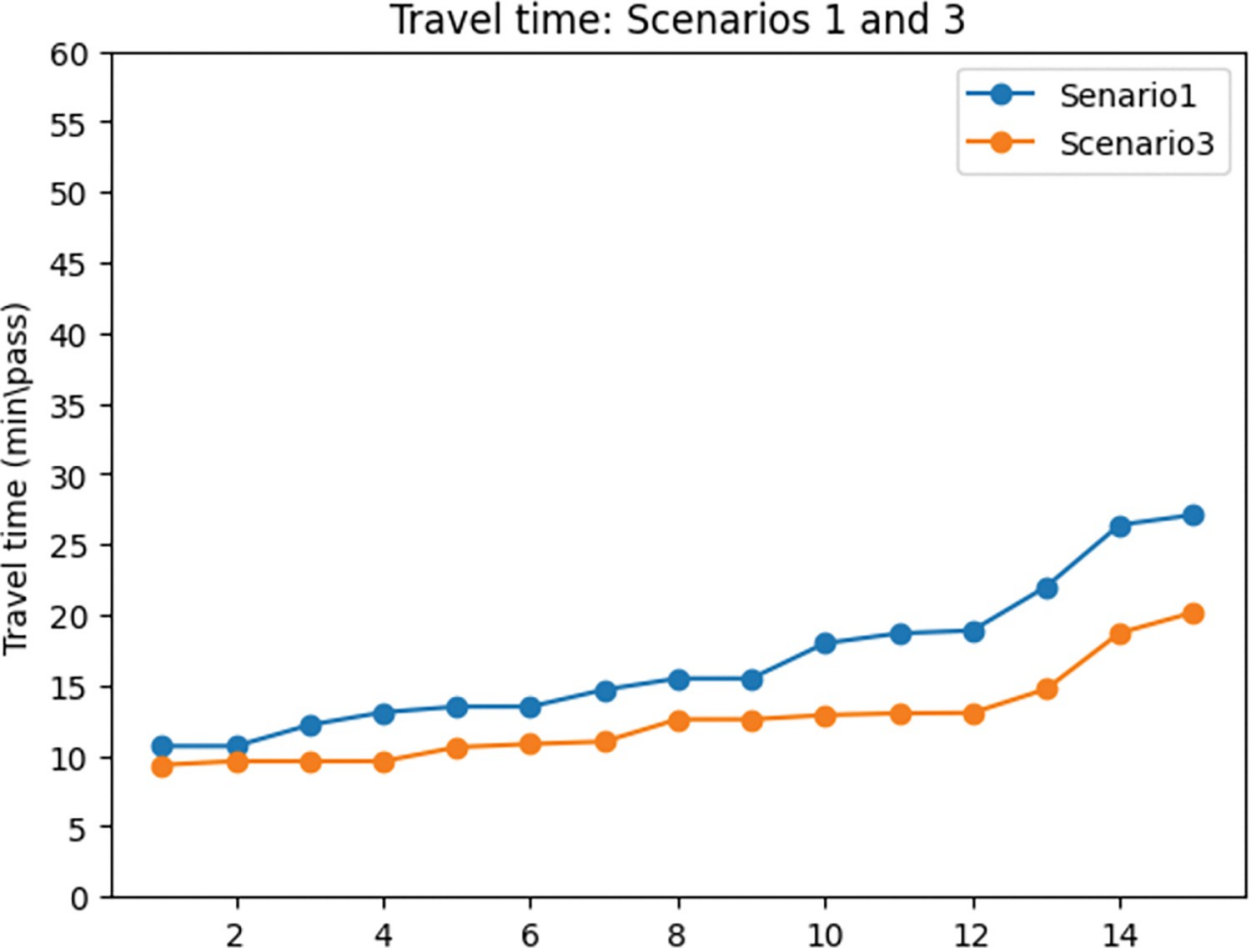
Travel time comparison between scenarios 1 and 3.

According to the Table 1, it is evident that incorporating the demand coverage objective function in the network design problem induces a significant increase in the proportion of total demand covered by the network. This finding is also demonstrated in Fig 12. As a result of the increase in demand coverage (47%), we expect travel time to increase, which is confirmed by Table 1 and Fig 13. Specifically, the results of the first and third scenarios reveal that when considering the demand coverage objective function in the model, the total travel time in the network increases by 28%.

**Fig 14:**
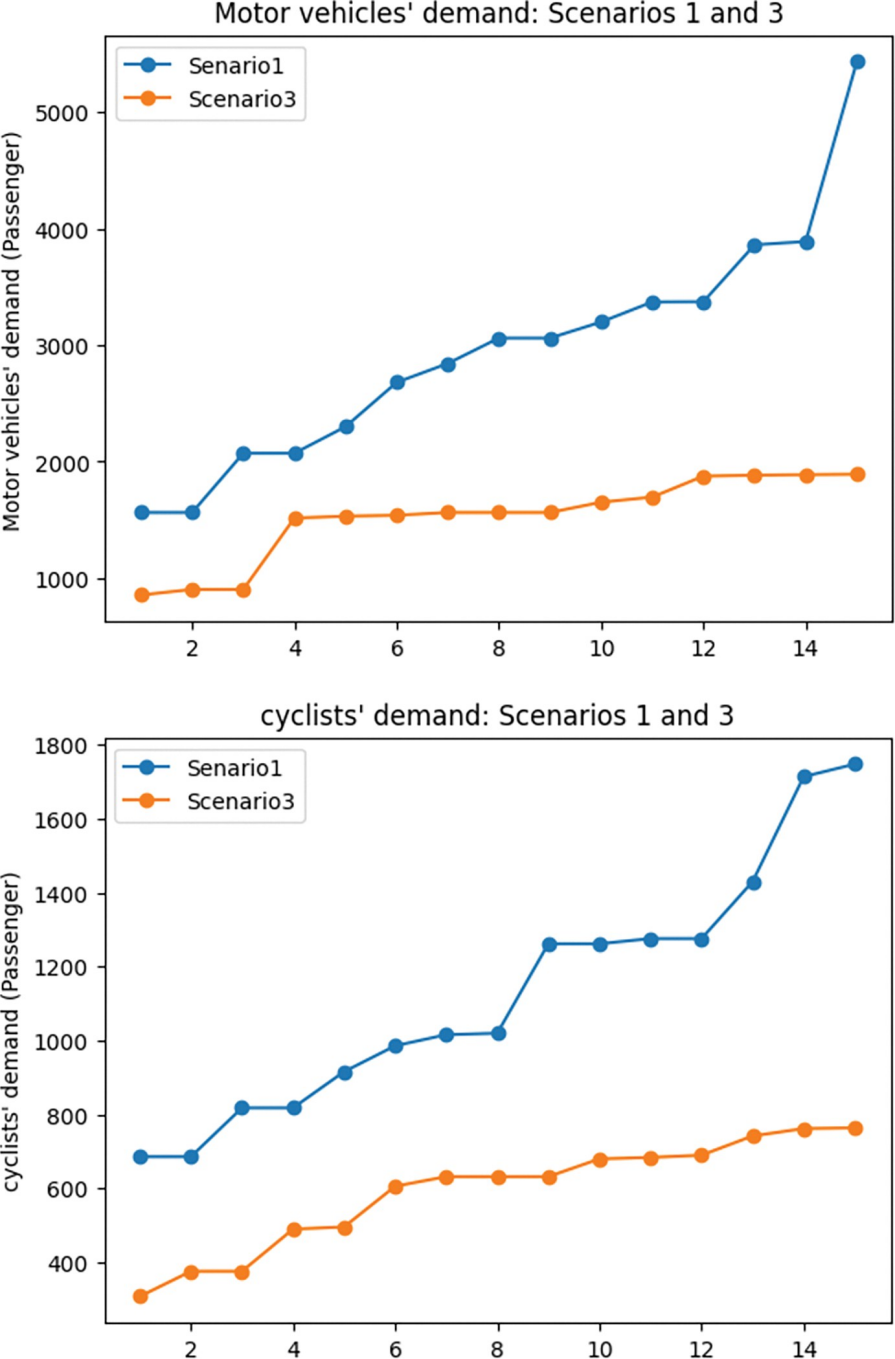
Considered modes’ demand coverage comparison between scenarios 1 and 3. a) Motor vehicles’ covered demand: scenarios 1 and 3. b) Cyclists’ covered demand: scenarios 1 and 3.

**Fig 15:**
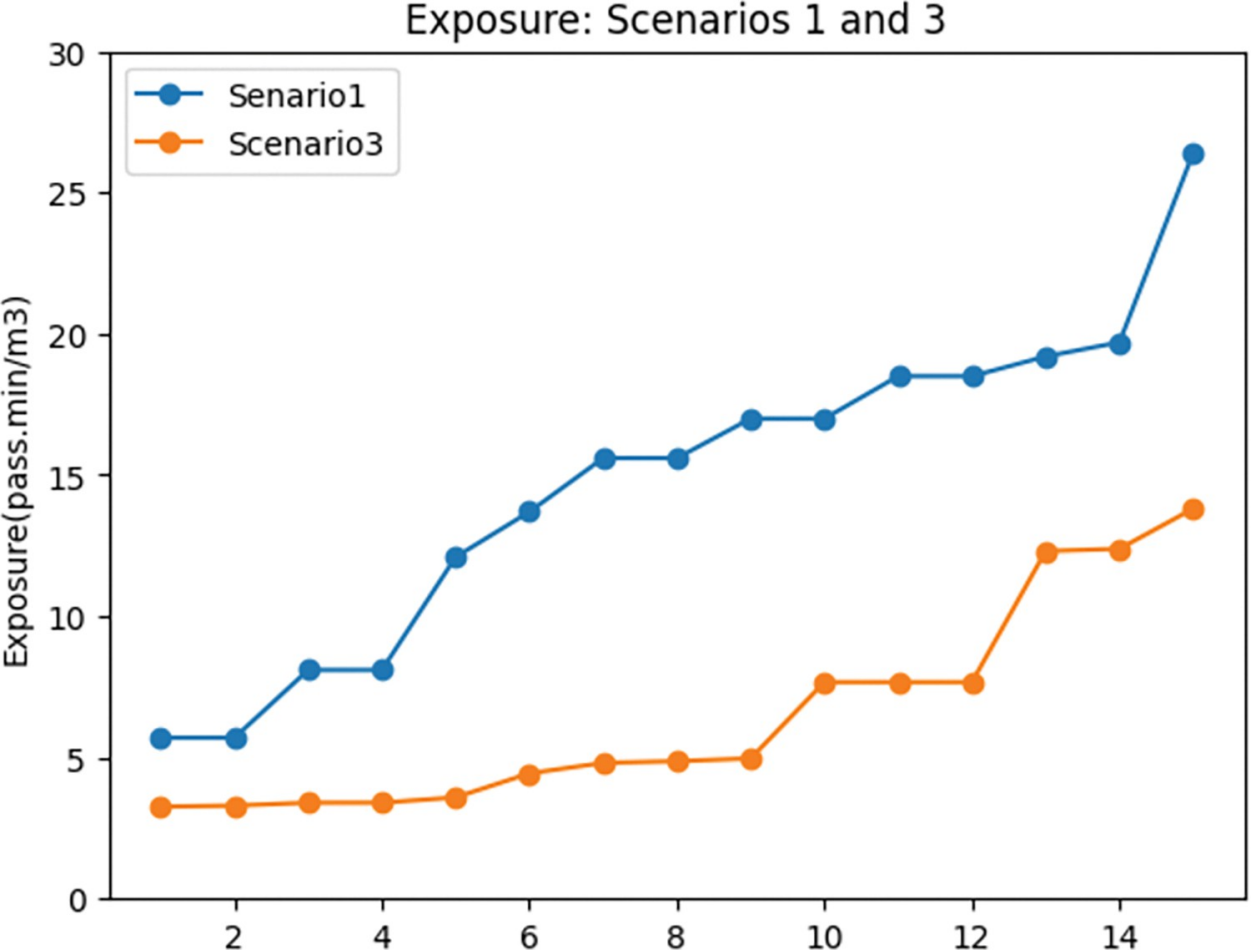
Exposure to traffic-generated air pollution comparison between scenarios 1 and 3.

In order to further examine the details related to demand coverage, Fig 14 illustrates the shift in motor vehicle demand and the cyclists’ demand in the first and second scenarios. It can be attended from the figure that the demand for motor vehicles and cyclists decreased in the third scenario. To be more specific, the motor vehicle demand experienced a 48% decrease in the third scenario, and cyclists’ demand shows a 47% drop in the third scenario. As a result of this decrease in demand for motor vehicle traffic, it is expected that the amount of pollutant emission and the exposure to traffic-generated air pollution for cyclists drops in the third scenario. Fig 15 dedicates the changes in the exposure objective in scenarios 1 and 3, which shows a 58% drop in exposure to traffic-generated air pollution in the third scenario.

Please note that the lines between the two markers do not represent real connectivity; rather, they are drawn to provide the reader with an intuitive sense of changes.

### Effects of exposure

One of the most important goals of the presented model is to design a cycling network to reduce exposure to traffic-generated air pollution. In order to study the effects of exposure to traffic-generated air pollution on the results of the model, scenarios 4 and 1 were compared.

**Fig 16:**
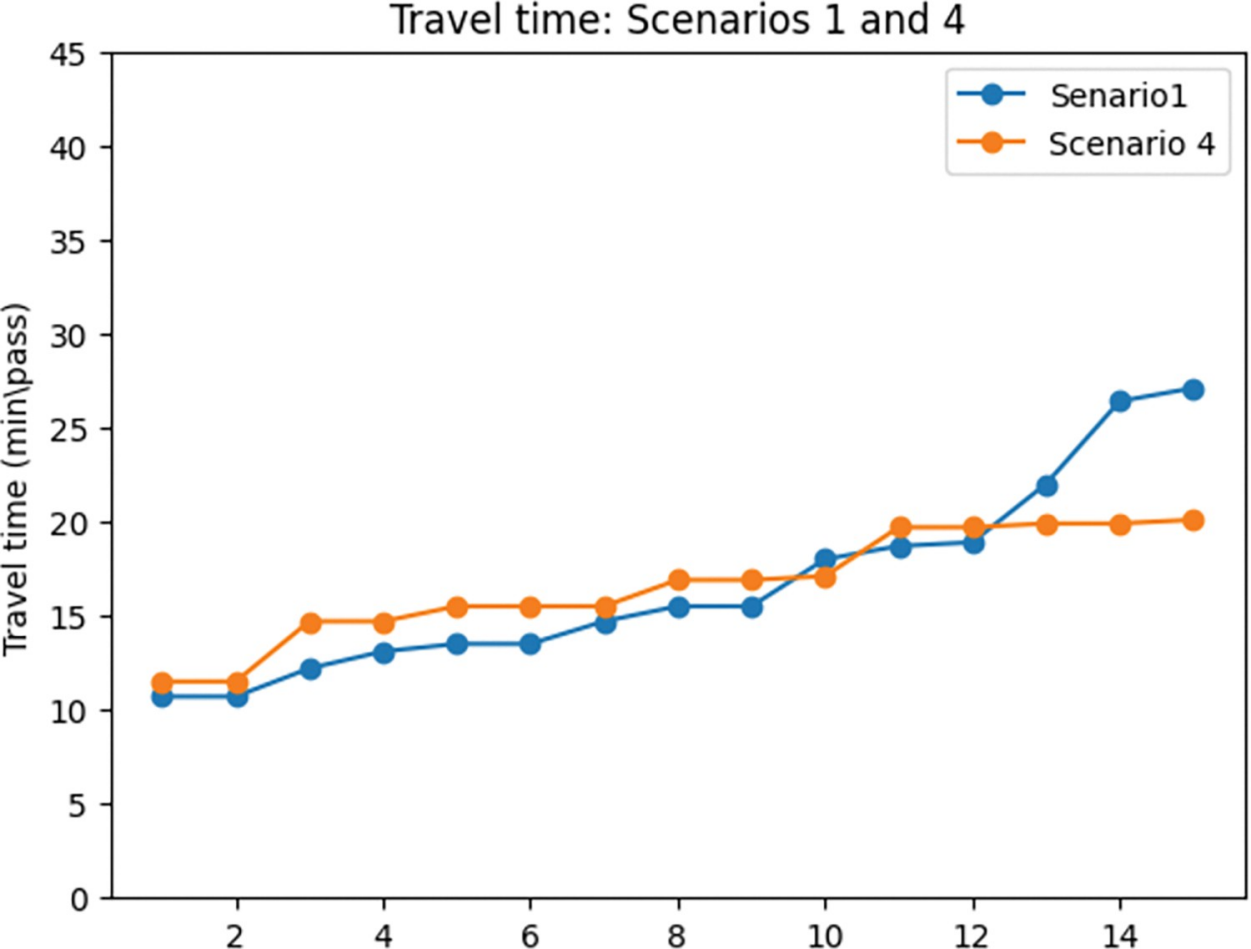
Travel time comparison between scenarios 1 and 4.

**Fig 17:**
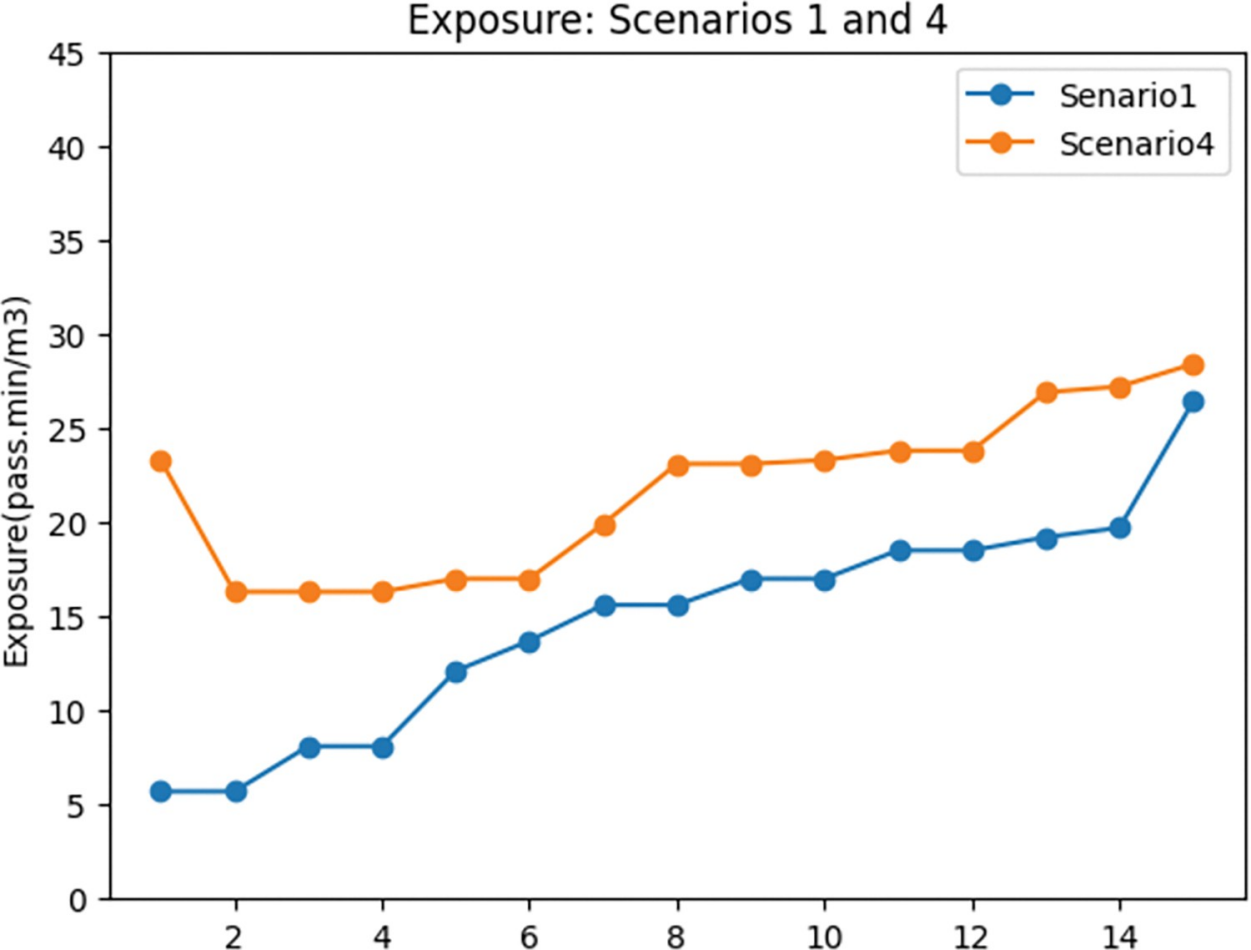
Exposure to traffic-generated air pollution comparison between scenarios 1 and 4.

**Fig 18:**
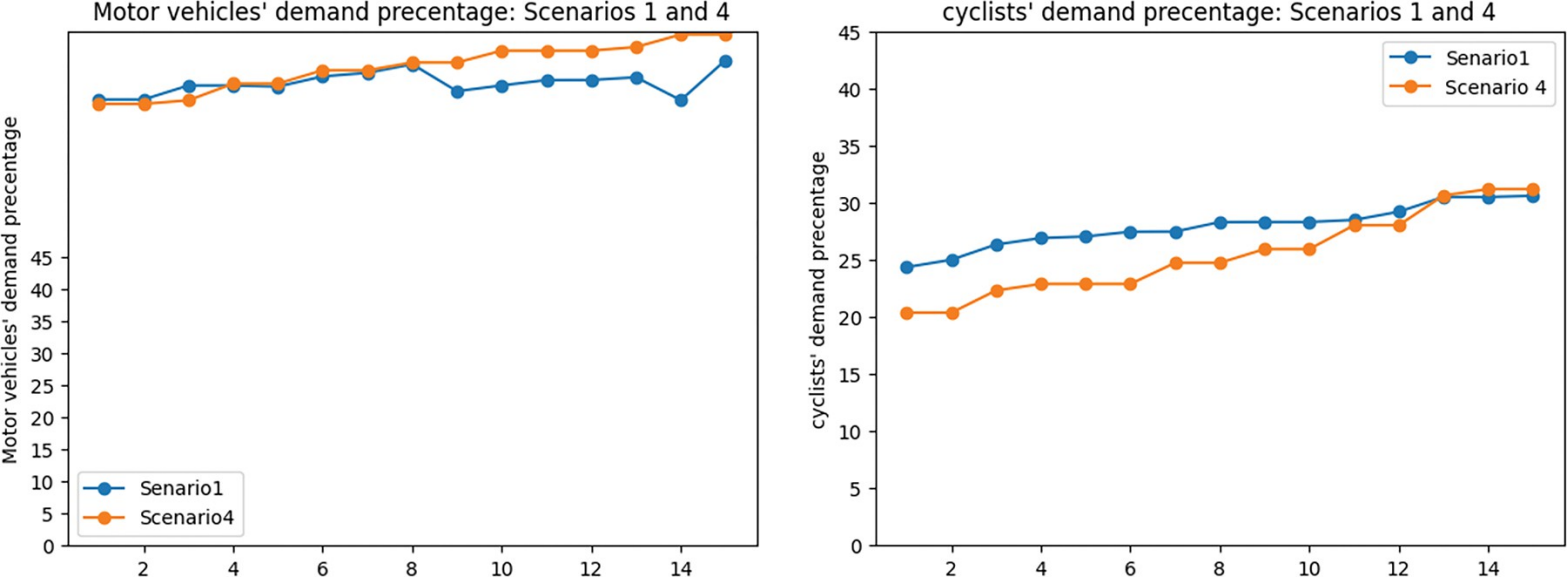
Motor vehicles’ and cyclists’ covered demand percentage 1 and 4. a) Motor vehicles’ covered demand percentage: scenarios 1 and 4. b) Cyclists’ covered demand percentage: scenarios 1 and 4.

Figs 16 and 17 show that adding exposure as an objective to the MMND model will results in a slight (4%) increase in total travel time in the network while decreasing exposure to traffic-generated air pollution (47%). Additionally, considering the exposure objective leads to a lower portion of total demand being covered by the network. To provide more details on these changes, the percentage of motor vehicles and cyclist demand compared to the total demand of the network was calculated and presented in Fig 18. According to this figure, in scenario 1, a higher portion of total demand (8.7%) was assigned to cyclists, while in scenario 4, the portion of motor vehicle demand is 3.4% higher compared to the first scenario. This increase in the portion of motor vehicle demand in scenario 4 and the decrease in cyclists’ portion results in higher exposure to air pollution. It is worth mentioning that the average total demand in scenario 4 is higher than in scenario 1 (by 22%). As we mentioned a higher portion of the covered demand in the fourth scenario is assigned to motor vehicles.

In this part, the percentage of car users and cyclists compared to the total demand in the network is presented as cars’ and cyclists’ demand respectively. The reason for using this concept is to provide a clearer understanding of changes in demand.

### Compare with a bicycle network design model from the literature

This subsection compares the results obtained from our model with those obtained from the model presented by Ospina et al. [8]. Since the case of exposure to traffic-generated air pollution has not been discussed in the network design problem in previous works, we select one of the relatively similar studies to our study in terms of objective functions. To compare the two models, we proposed a small network that is illustrated in Fig 7. This network was used due to its simplicity in comparison to larger networks and due to the possibility of assuming the specifications required for the model.

The model presented by Ospina et al. [8] is a bicycle network design problem that aims to maximize the coverage of cycling demand while considering the construction cost for the network. Ospina et al. [8] model is a single-level network design model that considers only cycling. Here, we aim to compare the literature model and our model to evaluate the effects of considering exposure and cost objective function in the network design model for cycling. To have a fair comparison, the demand coverage objective function in our model was considered only for cyclists.

We used the NSGA-II algorithm to solve both models. The algorithm chose 4 routes for the network each route has at least 3 links. We assumed that the construction cost is 3 units per *Km*. Table 2. represents the results of these two models. It is worth mentioning that the genetic algorithm has a probabilistic nature. As we used the same number of iterations for both models despite the differences in the number of objective functions, there might be a slight randomness. To address this issue, we run each model 5 times and chose the answer with the minimum exposure objective function for our model and the minimum value for the cost objective function in the literature model in each run. We used the average of the 5 answers that were achieved from each run.

**Table 2:** Optimal plans obtained from the literature and the presented model.

Model	Travel time	Exposure	Demand coverage	Cost
The Ospina et al. [8] model	27 (min/pass)	0.84 (pas.min.mg/m^3^)	28 passengers	10.08 (unit)
Our proposed model	24.16 (min/pass)	0.50 (pas.min.mg/m^3^)	28 passengers	16.8 (unit)

The table shows that the average covered demand for cycling in both models is the same. This indicates that the cost function or exposure objective function did not have a significant impact on the results for cyclists’ demand coverage. Another observation is that the travel time in our model is 10.5% lower than the travel time achieved by the Ospina et al. [8] model, which means that adding the exposure objective function led to better performance in terms of travel time. Our model achieved a 40.4% reduction in exposure to traffic-generated air pollution for cyclists compared to the literature model. Considering the exposure objective function helped the model choose less polluted links for cyclists. It is important to note that our model exceeded the budget limitation by 55%.

These results illustrate that considering exposure objective function leads to a network with a lower exposure for cyclists. However, budget is an important issue for authorities. To this end, the planners and decision-makers can decide which model to use according to their needs and priorities. It is also possible to use the combination of these models to take into account the trade-off between cost and exposure.

## Conclusion

The aim of our study is to design a cycling network that reduces cyclists’ exposure to traffic-generated air pollution by considering various objective functions. To achieve this, we formulate a bi-level multi-objective programming model that optimizes the cycling network design by taking into account travel time, exposure, and demand coverage, while considering exclusive bike and bus lanes. Cars, buses and cyclists were considered as transportation modes in this model. We used the non-dominated storing genetic algorithm and a method of successive average for the upper-level and the lower-level model respectively. We tested the model and the algorithm in a numerical example from the literature.

Our results demonstrate the effectiveness of the proposed model in designing cycling networks that prioritize the reduction of cyclists’ exposure to traffic-generated air pollution. The different tested scenarios, show the trade-off that needs to be made between different objectives. We found that involving exposure objective function and exclusive lanes for cyclists and buses in the network design problem have a significant impact on the reduction of exposure to traffic-generated air pollution with a slight increase in travel time. Furthermore, the demand coverage objective function has a positive effect on the increase of demand for all modes in the network. Finally, the results indicate that the proposed model outperforms the reference model in a small network in terms of reducing exposure to traffic-generated air pollution. However, there is a trade-off between the cost function that was considered in the literature model and the exposure function in our model. The cost function prioritizes reducing construction costs while the exposure function prioritizes reducing the health impacts of traffic-generated air pollution for cyclists. The decision on which function to prioritize depends on the specific goals of the urban planner in each city.

Further research can focus on involving other measures that affect the exposure of cyclists, for instance, the level of their activity in various parts of the network. Including other modes of transportation such as pedestrian and electronic bicycles will result in a more comprehensive evaluation of the whole transportation system. Considering emission and exposure as measures of the environmental capacity of roads and incorporating them as capacity constraints during the design stage can assist city planners in managing traffic-generated air pollution in the network.

## Supporting information

S1 File Appendix A: Links’ travel time for each exclusive lane type. (DOCX)
