# Supplementary material for: Cyclists’ exposure to traffic-generated air pollution in multi-modal transportation network design problem
Source: PLoS One. 2023 Jun 2;18(6):e0286153. doi: 10.1371/journal.pone.0286153 (PMC10237391; doi:10.1371/journal.pone.0286153)
Supplement: S1 File — (DOCX) [file pone.0286153.s001.docx]

**Appendix A: Links’ travel time for each exclusive lane type**

The bicycle width in exclusive lanes type 2 and 3 which are on-street lanes is assumed 1.5 meters. Considering the width of each lane is 3 meters, the exclusive lane with a width of 1.5 occupies 50% of the lane's capacity. Bike exclusive lanes type 4 and 5 which are off-street lanes are assumed to have a width of 2.5 meters. To simplify we supposed that these exclusive lanes occupy a lane. Existence of sidewalk for off-street is denied. Therefore, the BPR equation for each travel mode and each type of exclusive lane is presented as following:

Bicycle travel time

Exclusive lane type 1:

| (A.1) | $\forall a\in A$ $in.t_{a,1}^{b}=t_{0}^{b}\times[1+\alpha\times(\frac{x_{a}^{c}+x_{a}^{p}+x_{a}^{b}}{n_{a}\times C_{a}})^{\beta}]$  |
| --- | --- |

Exclusive lane type 2:

| (A.2) | $\forall a\in A$ $in.t_{a,2}^{b}=t_{0}^{b}\times[1+\alpha\times(\frac{x_{a}^{b}}{0.5\times C_{a}})^{\beta}]$  |
| --- | --- |

Exclusive lane type 3:

| (A.3) | $\forall a\in A$ $in.t_{a,3}^{b}=in.t_{a,2}^{b}$  |
| --- | --- |

Exclusive lane type 4:

| (A.4) | $\forall a\in A$ $in.t_{a,4}^{b}=t_{0}^{b}\times[1+\alpha\times(\frac{x_{a}^{b}}{C_{a}})^{\beta}]$  |
| --- | --- |

Exclusive lane type 5

| (A.5) | $\forall a\in A$ $in.t_{a,5}^{b}=in.t_{a,4}^{b}$  |
| --- | --- |

Bus travel time

Bus exclusive lanes are assumed to have width of 3 meters with is as wide as a lane.

Exclusive lane type 1:

| (A.6) | $\forall a\in A$ $in.t_{a,1}^{p}=t_{0}^{p}\times[1+\alpha\times(\frac{x_{a}^{c}+x_{a}^{p}+x_{a}^{b}}{n_{a}\times C_{a}})^{\beta}]$  |
| --- | --- |

Exclusive lane type 2:

| (A.7) | $\forall a\in A$ $in.t_{a,2}^{p}=t_{0}^{p}\times[1+\alpha\times(\frac{x_{a}^{p}}{C_{a}})^{\beta}]$  |
| --- | --- |

Exclusive lane type 3:

| (A.8) | $\forall a\in A$ $in.t_{a,3}^{b}=t_{0}^{p}\times[1+\alpha\times[\frac{x_{a}^{c}+x_{a}^{p}}{(n_{a}-0.5)\times C_{a}}]^{\beta}]$  |
| --- | --- |

Exclusive lane type 4:

| (A.9) | $\forall a\in A$ $in.t_{a,4}^{p}=in.t_{a,3}^{p}$  |
| --- | --- |

Exclusive lane type 5

| (A.10) | $\forall a\in A$ $in.t_{a,5}^{p}=t_{0}^{p}\times[1+\alpha\times[\frac{x_{a}^{c}+x_{a}^{p}}{(n_{a}-1)\times C_{a}}]^{\beta}]$  |
| --- | --- |

Car travel time

Exclusive lane type 1:

| (A.11) | $\forall a\in A$ $in.t_{a,1}^{c}=t_{0}^{c}\times[1+\alpha\times(\frac{x_{a}^{c}+x_{a}^{p}+x_{a}^{b}}{n_{a}\times C_{a}})^{\beta}]$  |
| --- | --- |

Exclusive lane type 2:

| (A.12) | $\forall a\in A$ $in.t_{a,2}^{c}=t_{0}^{c}\times[1+\alpha\times(\frac{x_{a}^{c}}{(n_{a}-1.5)\times C_{a}})^{\beta}]$  |
| --- | --- |

Exclusive lane type 3:

| (A.13) | $\forall a\in A$ $in.t_{a,3}^{c}=t_{0}^{c}\times[1+\alpha\times[\frac{x_{a}^{c}+x_{a}^{p}}{(n_{a}-0.5)\times C_{a}}]^{\beta}]$  |
| --- | --- |

Exclusive lane type 4:

| (A.14) | $\forall a\in A$ $in.t_{a,4}^{c}=t_{0}^{c}\times[1+\alpha\times(\frac{x_{a}^{c}}{(n_{a}-2)\times C_{a}})^{\beta}]$  |
| --- | --- |

Exclusive lane type 5

| (A.15) | $\forall a\in A$ $in.t_{a,5}^{c}=t_{0}^{c}\times[1+\alpha\times[\frac{x_{a}^{c}+x_{a}^{p}}{(n_{a}-1)\times C_{a}}]^{\beta}]$  |
| --- | --- |
